# Structure features of *Streptococcus pneumoniae* FabG and virtual screening of allosteric inhibitors

**DOI:** 10.3389/fmolb.2024.1472252

**Published:** 2024-09-27

**Authors:** Kaimin Xu, Jianliang Zhong, Jing Li, Yulu Cao, Lai Wei

**Affiliations:** ^1^ State Key Laboratory of Ophthalmology, Zhongshan Ophthalmic Center, Sun Yat-sen University, Guangzhou, China; ^2^ Molecular Cancer Research Center, School of Medicine, Shenzhen Campus of Sun Yat-sen University, Shenzhen, China; ^3^ State Key Laboratory of Oncology in South China, Sun Yat-sen University Cancer Center, Sun Yat-sen University, Guangzhou, China; ^4^ Guangdong Provincial Key Laboratory of Allergy & Clinical Immunology, The Second Affiliated Hospital, Guangzhou Medical University, Guangzhou, China; ^5^ Department of Ophthalmology, The First Affiliated Hospital, University of South China, Hengyang, China

**Keywords:** *Streptococcus pneumoniae*, FabG, virtual screen, fatty acid metabolism, anti-biotics

## Abstract

*Streptococcus pneumoniae*, a gram-positive bacterium, is responsible for diverse infections globally, and its antibiotic resistance presents significant challenges to medical advancements. It is imperative to employ various strategies to identify antibiotics. 3-oxoacyl-[acyl-carrier-protein] reductase (FabG) is a key component in the type II fatty acid synthase (FAS II) system, which is a developing target for new anti-streptococcal drugs. We first demonstrated the function of SpFabG *in vivo* and *in vitro* and the 2 Å SpFabG structure was elucidated using X-ray diffraction technique. It was observed that the NADPH binding promotes the transformation from tetramers to dimers in solution, suggesting dimers but not tetramer may be the active conformation. By comparing the structures of FabG homologues, we have identified the conserved tetramerization site and further confirmed the mechanism that the tetramerization site mutation leads to a loss of function and destabilization through mutagenesis experiments. Starting from 533,600 compounds, we proceeded with a sequential workflow involving pharmacophore-based virtual screening, molecular docking, and binding energy calculations. Combining all the structural analysis, we identified L1, L2 and L5 as a promising candidate for SpFabG inhibitor, based on the most stable binding mode in comparison to other evaluated inhibitors.

## Introduction

*Streptococcus pneumoniae*, a gram-positive bacterium, primarily colonizes the mucosal surfaces of the human upper respiratory tract in the form of biofilms and has globally resulted in a variety of infections (Li et al., 2023). It is the major bacterial cause of a wide range of infections, such as otitis media, community-acquired pneumonia, sepsis, and meningitis, with invasive inflammatory diseases occurring due to the local spread, aspiration, or seeding of S. pneumoniae to the bloodstream (Bogaert et al., 2004). The World Health Organization recognized the continued high burden of S. pneumoniae-associated diseases and its increasing antibiotic resistance, prompting its addition to the list of 12 priority pathogens in 2017. The WHO’s 2022 report highlighted the escalating public health threat of antimicrobial resistance, referred to as a “silent pandemic.” Nearly 4.95 million deaths per year are attributed to drug-resistant bacterial infections, with 1.27 million directly linked to resistance. In response to these challenges, efforts are being made to prevent infections, including vaccination, improved hygiene practices, appropriate use of antibiotics, and the development of novel antibiotics. However, antimicrobial resistance (AMR) is a slow-moving menace that threatens to reverse centuries of medical progress. S. pneumoniae has developed resistance to multiple antibiotics, such as penicillin, macrolides, fluoroquinolones, and sulfamethoxazole-trimethoprim, leading to treatment failures and increased morbidity and mortality rates (Prevention, 2024).

A common lifestyle for S. pneumoniae is its growth in biofilms, wherein aggregated communities produce and secrete a protective extracellular matrix including phospholipids (Yan and Bassler, 2019). Biofilm formation follows asymptomatic colonization in the nasopharynx (Valente et al., 2021). Notably, biofilms exhibit inherent tolerance to antibiotics, and some develop resistance due to multiple factors (Ciofu et al., 2022; Dincer et al., 2020). Recent studies have reported that the formation of pathogenic microbial biofilms and the targetability of cell membranes are regulated by fatty acid metabolic pathways (Boudjemaa et al., 2018; Arjes et al., 2022; Yao et al., 2022). Therefore, integrating current antibacterial methods with interventions targeting the bacterial fatty acid metabolic pathway could potentially offer a new antibacterial strategy. The *de novo* synthesis of fatty acids is a crucial biochemical pathway that is responsible for generating lipids, essential for cell membrane formation, biofilm construction, and serving as an intracellular energy source (Chao et al., 2014; Ohlrogge and Browse, 1995; Beld et al., 2015). The process is classified into two types, FAS I and FAS II system, based on the molecular machine arrangement. FAS II, primarily found in bacteria, plants, and parasites, involves the collaborative action of several individual enzymes in catalyzing fatty acid synthesis. Conversely, most mammals utilize the FAS I system, comprising a single large, multi-functional protein. This disparity makes FAS II an attractive drug target, leading to the use of antibiotics such as triclosan or isoniazid (Campbell and Cronan, 2001; Heath and Rock, 2004; Pan and Tonge, 2012; Wang and Ma, 2013; Yao and Rock, 2017). One critical component of FAS II system is 3-oxoacyl-[acyl-carrier-protein] reductase (FabG), which plays a vital role in the elongation process necessary for the production of both saturated and unsaturated fatty acids, utilizing NADPH to catalyze the initial reduction step, facilitating the conversion of 3-oxoacyl-ACP to 3-hydroxyacyl-ACP intermediates (Shanbhag, 2019a). Pucci et al. utilized gene knockout techniques to identify essential genes in *Streptococcus pneumoniae*, including fabG (Thanassi et al., 2002). Recent research has identified fabG of S. pneumoniae as an essential gene through CRISPRi assay (Liu et al., 2017), indicating that SpFabG may be a promising target for the development of anti-S. pneumoniae agents. Despite the potential, there are limited reports of SpFabG inhibitors.

Since the 1970s, the advancement of computerized screening of chemicals as potential ligands to a target protein, whether it belongs to a general category or is unique, has been continuous. The aim of this approach is to complement or even replace the laborious empirical process of *in vitro* screenings. Over time, virtual screening methods have evolved, leveraging vast digital libraries of compounds with drug-like properties and specific 3D receptor structures to achieve successful outcomes (Shoichet, 2004). The aspiration to establish virtual screening as a standalone methodology had been long-held. As computational chemistry modules have improved in accuracy and reliability, molecular docking simulations have become an integral part of the concept of “virtual screening” (Cheng et al., 2012a). Structure-based virtual screening workflows now incorporate Molecular Dynamics (MD) simulations, which simulate the behaviors of any given structure over nanoseconds using mathematical force-field models that define atomic interaction parameters (Dror et al., 2012). This enables a more flexible representation of the structure, leading to enhanced precision in eliminating compounds during virtual screening protocols (Batool et al., 2019). In the context of drug discovery targeting SpFabG, the methods and workflows mentioned above were replicated. A virtual screen of more than 500 thousand compounds based on the experimental SpFabG structure was performed. Subsequently, the high score docking results were further evaluated by binding free energy calculation and molecular dynamic simulations, leading to the identification of one hit as a promising inhibitor of SpFabG.

The superfamily of short-chain dehydrogenases/oxidoreductases (SDRs), to which FabG belongs, is highly conserved across various species and fabG gene has been identified as essential gene in many microorganisms (https://tubic.org/deg/public/index.php), including *Staphylococcus aureus*, *Mycobacterium tuberculosis*, *Pseudomonas aeruginosa*, *Escherichia coli* and so on. Inhibitory activities against FabG have been identified in plant-derived polyphenols epigallocatechin gallate (Zhang and Rock, 2004), which can reduce the minimum inhibitory concentration (MIC) of the antibiotics by fourfold in combination with antibiotics (Soe et al., 2010; Stapleton et al., 2004). Specific inhibitors of *Pseudomonas aeruginosa* FabG (PaFabG) have been shown to have significant inhibitory effects, with reported IC50 values in the nanomolar to low-micromolar range (Cukier et al., 2013). The potential of FabG inhibitors as broad-spectrum antibiotics underscores the importance of gaining a deeper understanding of the molecular-level reaction mechanisms and structure conservative site of bacterial FabGs for rational drug design purposes.

## Materials and methods

### Strains and growth conditions

We used the *E. coli* fabG temperature-sensitive (Ts) mutant strain CL104 (Lai and Cronan, 2004), *Saccharomyces cerevisiae* growth defective strain BY4741 and its derived strain BY4741oar1Δ to authenticate *Streptococcus pneumoniae* FabG’s function. *E. coli* strain CL104, generously provided by professors John E. Cronan from University of Illinois and Haihong Wang from South China Agricultural University, was cultured in Luria-Bertani (LB) medium at dedicated temperature with additional antibiotics and inducer as follows: sodium ampicillin, 100 μg/mL; chloramphenicol, 30 μg/mL; kanamycin sulfate, 100 μg/mL; tetracycline, 10 μg/mL; L-arabinose, 0.1% (w/v). Yeast strain BY4741 was a gift from professor Jing Li form Sun Yat-Sen University Cancer Center, and its derived strain BY4741oar1Δ was constructed through chemical transduction. Generally, DNA sequences comprising 500 bp upstream of oar1 gene, the ura3 promoter, ura3 gene, and 500 bp downstream of oar1 gene were synthesized and then amplified through PCR. Subsequently, the yeast transformation were carried out following the method proposed by Gietz (Gietz and Schiestl, 2007). Successful gene editing was indicated by the ability to grow on Synthetic Complete (SC) dropout medium without uracil (SC-URA) at 30°C and further confirmed by DNA sequencing. Phenotype of the transformants was observed to be growth defective in SC-glycerol medium containing 3% (w/v) glycerol. Other yeast medium used in the experiment included SC-glucose medium containing 2% (w/v) glucose and SC dropout medium without histidine (SC-HIS).

### Complementary assay of FabG activities *in vivo*

The potential of SpFabG to restore fatty acid synthesis was evaluated in the *E. coli* fabG(Ts) mutant strain CL104 (Lai and Cronan, 2004) as well as the *S. cerevisiae* strains BY4741 and BY4741oar1Δ. In the CL104 strain system, an arabinose-inducible vector pBAD24, carried FabG gene from S. pneumoniae or other species, was introduced into the CL104 strain. These strains carrying pBAD24 (with or without fabG) was induced with 0.1% (w/v) arabinose at a permissive temperature (30°C), or a non-permissive temperature (42°C). In the BY4741 strains system, the homologous proteins of FabG from *Streptococcus pneumoniae* and other species was cloned into the glycerol-inducible expression vector pCTA1HIS, which is modified from pESCHIS by replacing its GAL1-GAL10 promoter with CTA1 promoter and adding a downstream FLAG tag for detecting the production of the recombinant protein, and then transformed into oar1Δ yeast as Gietz described (Gietz and Schiestl, 2007). Yeast cultures were grown on SC-HIS plate, with only successfully transformed yeast capable of growth on the medium, resulting in visible colonies after 4 days. Transformants were diluted in PBS at a 10-fold gradient and then dispensed as 1.5uL aliquots onto SC-Glucose complete medium and SC-Glycerol selective medium. Photos were taken after 2 days of growth on SC-Glucose and after 4–5 days of growth on SC-Glycerol. The full scan of the entire plate photograph is shown in Supplementary Figures S1A, B, D.

### Sequence identification and multiple sequence alignment of FabGs

Amino acid sequences of FabG and homologs used in the multiple sequence alignment included *Streptococcus pneumoniae* FabG (UniProt accession Q8DR15), Clostridioides difficile FabG (Q18B45), *S. aureus* FabG (Q6G9Y2), *Pseudomonas aeruginosa* FabG (O54438), *Haemophilus* influenzae FabG (P43713), *E. coli* FabG (P0AEK2), *S. cerevisiae* FabG (P35731) and *Homo sapiens* FAS (P49327). The amino acid sequences of these eight FabG homologs were obtained from UniProt (UniProt Consortium, 2023) and aligned to identify conserved residues throughout the protein’s molecular evolution using EBI-MUSCLE (Li et al., 2015). The alignment was then visualized via ESpript (Robert and Gouet, 2014). We also constructed a phylogenetic tree of these eight FabG homologs using the Maximum Likelihood (Tamura et al., 2011) method with 1,000 bootstrap repetitions to assess their evolutionary divergence.

### Construct, protein expression and purification

The *E. coli* BL21 (DE3) pLysS cells were cultured until reached at an OD600 of 0.6 at 37°C, then induced overnight at 18°C with 0.1 mM isopropyl-1-thio-β-D-galactopyranoside (IPTG) for the production of a recombinant protein containing an N-terminal His6 tag followed by a PreScission protease (PSP) cleavage site. Subsequently, the cells were centrifuged at low speed to pellet, then resuspended in a lysis buffer containing 50 mM HEPES pH 7.5, 400 mM sodium chloride, and 30 mM imidazole, 1 µM DNase I, 1 mM phenylmethanesulfonyl fluoride (PMSF) and 2.5 mM β-mercaptoethanol (β-ME). Next, the cells were disrupted once using a cell disruptor (JNBIO) at 4°C and 1,000 bar pressure, followed by centrifugation at 50,000 g for 1 h at 4°C. The resulting supernatant was filtered and loaded onto a Ni-NTA column (GE healthcare) equilibrated with Binding Buffer 1 (20 mM HEPES pH 7.5, 400 mM NaCl, 30 mM imidazole and 2.5 mM β-ME). The bound proteins were eluted using Elution buffer containing 20 mM HEPES pH 7.5, 400 mM NaCl, 300 mM imidazole and 2.5 mM β-ME, after being washed with Binding Buffer 1. Following this step, the His6-tag was removed by incubating the eluted proteins with 2 µg GST-fused PSP. The His6-tag free proteins were then dialyzed overnight against Binding Buffer 2 (20 mM HEPES pH 7.5, 400 mM NaCl and 2.5 mM β-ME) and subjected to a GST column to remove PSP. The proteins were subsequently reapplied to a second Ni-NTA column equilibrated with Binding Buffer 2 and were eluted with Binding Buffer 1. Finally, the eluted proteins were loaded onto a Superdex 200 16/600 column (GE healthcare) equilibrated with Gel Filtration Buffer containing 20 mM HEPES pH 7.5, 250 mM NaCl and 1 mM dithiothreitol (DTT). The protein concentration was initially determined using a BCA protein assay kit (ThermoFisher), and the results were recorded. The proteins were temporarily stored at 4°C and then snap-frozen in liquid nitrogen for long-term preservation.

### Protein crystallization and structure determination

In the crystallization experiment, we adjusted the protein concentration to 15 mg/mL and screened hundreds of conditions including JCSG II, PEG ION, etc., using the sitting-drop method and the protein was mixed with solution in a 1:1 ratio, and crystal growth was observed under various conditions. Detailed crystallization conditions are summarized in Supplementary Table S1. In the optimization stage, the crystals were cultivated at 4°C under a 1:1 mixing condition of 15 mg/mL protein with 0.1M Acetate pH 4.5% and 40% v/v 1,2-propanediol. The diffraction data were collected at beamline BL17U1 of the Shanghai Synchrotron Radiation Facility (SSRF). Subsequently, all data underwent processing using the XDS (Kabsch, 2010) software, followed by further refinement with the CCP4 suite (Agirre et al., 2023) and the Phenix suite (Adams et al., 2010). Molecular replacement was executed by Phaser (McCoy et al., 2007), employing a search model built with AlphaFold (Jumper et al., 2021). Refinement was conducted using Phenix. refine. The structural validation was conducted through MolProbity (Davis et al., 2007). To illustrate the structure, PyMol (DeLano, 2002) was employed. Detailed X-ray data collection and refinement statistics are available in Table 1. Notably, the Ramachandran statistics determined by MolProbity demonstrate that 97.68% of the structure is within the favored region, with 2.32% being allowed, and no outliers.

**TABLE 1:** Crystallographic data collection and refinement statistics.

State	FabG
Data collection	
Space group	P212121
Cell dimensions	
a, b, c (Å)	67.71, 125.27, 127.25
α, β, γ (°)	90
Wavelength (Å)	0.979,183
Resolution (Å)	56.73–2.06 (2.11–2.06)
R_merge_	0.149 (1.809)
R_pim_	0.062 (0.757)
CC(1/2)	0.996 (0.690)
I/σ(I)	10.7 (2.49)
Completeness (%)	99.7 (100.0)
Redundancy	12.5 (13.0)
Refinement	
Resolution (Å)	56.73–2.06
No. reflections	67,490
R_work_/R_free_	0.211/0.248
No. atoms	
Protein	6,823
Ligand/ion	0
Water	312
B-factor	
protein	47.8
Water	48.6
R. m s deviations	
Bond lengths (Å)	0.009
Bond angles (°)	1.14
Ramachandran plot	
Favored (%)	97.68
Allowed (%)	2.32
Outliers (%)	0
MolProbity score	1.49
clashscore	7.75

### SDS-PAGE analysis

The protein sample was first mixed with 5X sample buffer and incubated at 100°C for 5 min. Following this, the processed samples and protein marker were separated on 12% (w/v) polyacrylamide gels, which were then stained with Coomassie Blue dye and de-stained with hot water. This process was followed by capturing gel images using a ChemiDoc Imaging System (Bio-Rad) as the gel background was clarified.

### Analytical size-exclusion chromatography

Superdex 200 Increase 10/300 GL column was used for analytical size-exclusion chromatography (SEC). After incubating 200 μL of 1 mg/mL SpFabG with 5 mM NADPH at 4°C for 2 h, the mixture was centrifuged at 100,000 x g for 10 min, and the resulting supernatant was used for further analysis. The SEC was conducted at 4°C using a buffer containing 20 mM HEPES pH 7.5, 150 mM NaCl, and 1 mM DTT, with a flow rate of 0.5 mL/min and the absorption at 280 nm was recorded.

### Western blot analysis

Yeast colonies from SC-glucose (SCD) plate were homogenized in Radio-Immunoprecipitation Assay (RIPA) buffer (#P0013B, Beyotime, China) on ice fortified with protease inhibitors cocktail (#B14001, Bimake, United States) to prevent unwanted enzymatic degradation. After 2 h, the lysates were centrifuged to obtain the supernatant, which was quantified for protein content using the Bicinchoninic Acid (BCA) assay (#23225, Thermo Scientific, United States).

Protein samples were resolved via SDS-PAGE and subsequently transferred onto a 0.22 μm PVDF membrane. The membranes were incubated with antibodies (#293881 Mouse anti-Flag, Abmart, China) and (#M30109 Mouse anti-beta-tubulin, Abmart, China) overnight at 4°C. After that, secondary antibodies (#M21002 Goat Anti-Rabbit IgG-HRP, Abmart, China) was applied and gently shaken at room temperature for 1 h. Protein samples were visualized using an ECL chemiluminescence kit (#34577 SuperSignal West Pico PLUS, Thermo Scientific, United States) as shown in Supplementary Figure S1C.

### 3‐oxoacyl‐ACP reductase (OAR) activity assay *in vitro*

OAR activity assays for SpFabG were carried out in 100 mM sodium phosphate (pH 7.0), 1 mM acetoacetyl CoA (AcAcCoA) and 1 mM NADPH. Monitoring the consumption of NADPH was conducted with an Agilent BioTek Synergy H1 plate reader in absorbance at 340 nm. To facilitate the experimental measurements, the equation relating NADPH concentration to the 340 nm absorbance was predetermined, allowing the conversion of absorbance values to NADPH concentrations during the experiment. The reactions, tailored to the unique requirements of each reagent, were set up in 100 μL total volume within 96-well plates. The FabG protein was introduced to initiate the reactions, and NADPH consumption was monitored over a 5-min period by measuring the decrease in absorbance at 340 nm. The enzyme activity data were analyzed using linear regression, with the resulting slope representing the average reaction rate.

### Statistical analysis

Statistical analysis was conducted using SciPy package, with results presented as mean ± SD. Statistical significance levels were defined as ^*^ *p* < 0.05, ^**^ *p* < 0.01, ^***^ *p* < 0.001, and ^****^ *p* < 0.0001. Group comparisons were performed using a one-way analysis of variance (ANOVA).

### Virtual screening, binding energy calculation and ADMET assessment

The SpFabG tetramer underwent refinement through the removal of water molecules and then optimized and energy minimized using the Schrodinger protein preparation wizard module, for hot spot identification and virtual screening. We used two methods, Geometry-based CB-Dock2 (Liu et al., 2022) and grid-based DoGSiteScorer (Volkamer et al., 2012), to predict binding hot spot at the dimer interface A individually and both methods correctly identified the same binding site. Considering the hydrophobic nature of the binding site and limited space, we retrieved a dataset containing 533,600 small molecules from the ZINC (Irwin et al., 2012) that met the criteria of molecular weight <250, 0<logP <5, in-stock availability, and neutral charge. Prior to commencing docking studies with target molecules, the molecular format was readied using Schrodinger LigPrep module. The OPLS2005 force field was employed to enhance the structure of the identified ligand. Just as in protein preparation, tautomers and stereoisomers were manipulated during ligand preparation to simplify the geometric intricacies. Pharmacophore models were constructed by PHASE (Dixon et al., 2006) using the receptor cavity model and e-pharmacophore method, with the geometric centers of 208M, 216Q, and 237I defined as receptor binding sites, while other parameters were set to default values. During molecular docking with Glide (Friesner et al., 2006), a cubic docking box of 12 Å was employed, with all other parameters maintained at their default settings. Based on the docking scores, the binding free energy of the top 1% ligands was calculated using Schrodinger Prime. During the docking procedures, the flexibility of only the ligands was taken into account, while the structure of the SpFabG protein was held rigid. Finally, based on binding free energy ranking, docking posture of the top 5 compounds were visually examined, and their physicochemical and pharmacokinetic ADMET parameters were detailed assessed via ADMETlab (Xiong et al., 2021).

### Construction and validation of complete protein model

Because NADPH binding site containing region β4-α4 loop is highly flexible and the electron density of the corresponding region is not visible, this amino acid segment is missing from the final model. To obtain the full-length protein structure for further study, the 3D structure of chimeric SpFabG was modeled using homology modeling tools MODELLER (Webb and Sali, 2016). The amino acid sequence of SpFabG was initially scanned against the PDB (Berman et al., 2000)database using the integrated PSI-BLAST algorithm of the NCBI (Altschul et al., 1997)to determine a homology modelling template. To ensure the structural validity of the SpFabG model, qualitative evaluations were conducted using various structure validation servers. The integrity of torsion angle conformations within SpFabG was evaluated using UCLA-DOE’s server (https://saves.mbi.ucla.edu/) and its Ramachandran plot. The output scores from these servers were then compared against parameters of authentic 3D structures analyzed by experimental methods. ProSA (Wiederstein and Sippl, 2007)was utilized to assess whether the overall structure of the SpFabG model was correctly energy optimized, allowing for a comprehensive evaluation of the accuracy and reliability of the model, providing valuable insights into the quality of its 3D structure.

### Molecular dynamics simulation and trajectories analysis

The top 5 complexes identified by MM/PBSA binding energy calculation were further validated through a 200 ns molecular dynamics simulation, conducted using GROMACS (Kutzner et al., 2015). The initial structures for the MD simulations were derived from the representative docking pose obtained from molecular docking results. The protein was modeled with the Amber force field (Tian et al., 2020), and the ligands were modeled using the GAFF force field (Wang et al., 2004). To solvate each system, a rectangular water box was employed, and a 10 Å buffer was maintained between the proteins and the box edge, with the TIP3P model (Jorgensen et al., 1983) employed for the water molecules. Neutralization of the systems was achieved by adding counter ions (0.15 M NaCl), followed by a steepest descent energy minimization (∼5,000 steps). Subsequently, the systems were equilibrated in the NVT and NPT ensembles for 500 ps each, using the LINCS method (Hess, 2008) to constrain all bond lengths with a 2 fs time step. The temperature and pressure were set at 303 K and 1 atm, respectively, using the Nose-Hoover thermostat (Hoover, 1985; Nosé, 1984) and the Parrinello-Rahman barostat (Parrinello and Rahman, 1981). The long-range electrostatic interactions were computed utilizing the particle mesh Ewald algorithm (PME) (Darden et al., 1993), and a 1.4 nm cut off was applied for short-range van der Waals interactions. Snapshots of the MD trajectories were saved at intervals of 20 ps for subsequent analysis.

After MD simulation, we evaluated the stability of proteins and inhibitors by measuring the root-mean-square deviation (RMSD), which is a measurement of how much the structure deviates from its reference state, away from their initial structures. The RMSD of the protein and ligand are calculated using the protein backbone atoms and all atoms of the ligand, respectively. We also investigated the hydrogen bonds between the protein and ligand, that formed when a hydrogen atom shares its electrons with two other atoms and could help the protein and ligand bind more specifically and effectively. VMD (Humphrey et al., 1996) plugin Hydrogen Bonds was used to judge whether the hydrogen bonds were formed during simulations using specific rules that a hydrogen bond formed when the distance between the hydrogen atom and acceptor atom was less than 3.5 A and the angle between the donor atom, hydrogen atom, and acceptor atom was greater than 150°. In total, we analyzed 10,000 images from each 200 ns simulation after removing any rotational or translational movements and finally visualized the MD trajectories using VMD.

## Result

### *S. pneumoniae* FabG complemented the growth of the *Escherichia coli* and yeast *in vivo*

While several studies have identified FabG as an essential gene for *Streptococcus pneumoniae* and crucial for its survival (Thanassi et al., 2002; Liu et al., 2017), research on SpFabG as an antibiotic target is currently lacking. With the rising demand for new antibiotics, more researchers are turning their attention to the FabG enzyme in the bacterial FAS II fatty acid synthesis system. First, we performed a BLAST search of the SpFabG protein sequence in the *Streptococcus* pneumoniae reference database, which resulted in only one hit, the SpFabG itself, indicating that SpFabG has only one copy in the genome (data not shown). To assess the putative 3-oxoacyl-[acyl-carrier-protein] reductase functions of S. pneumoniae FabG *in vivo*, we constructed S. pneumoniae FabG and other functional fabG genes into the pBAD24 vector under the arabinose-regulated araBAD promoter, allowing for an induced transcription of the foreign genes. Plasmids were transformed into the *E. coli* fabG (Ts) strain CL104 (Lai and Cronan, 2004) at 30°C to grow and the resulting transformants were evaluated for their ability to grow on rich broth (RB) plates at the nonpermissive temperature of 42°C. It was found that the derivatives of CL104 carrying pBAD24-SpFabG and other functional FabGs exhibited robust growth at 42°C in the presence of arabinose, while the CL104 carrying the empty vector did not show this capacity (Supplementary Figure S2A). The subsequent assay was conducted to evaluate the 3-oxoacyl-[acyl-carrier-protein] reductase activity within eukaryotic cells. The FabG candidates were subcloned into the yeast mitochondrial localization vector pCTA1HIS and subsequently assessed for complementation in the yeast oar1Δ strains. The production of SpFabG in yeast resulted in the rescue of respiratory deficiency in oar1Δ strains, as depicted in Supplementary Figure S2B. The strains carrying plasmids producing either functional FabGs or the yeast OAR1 exhibited the same growth as the wild-type strains. These demonstrated that SpFabG has the capability to complement both *E. coli* and yeast *in vivo*, thereby functioning as 3-oxoacyl-[acyl-carrier-protein] reductase FabG.

### Sequence retrieval and assessment of sequence similarity among FabG species

**FIGURE 1:**
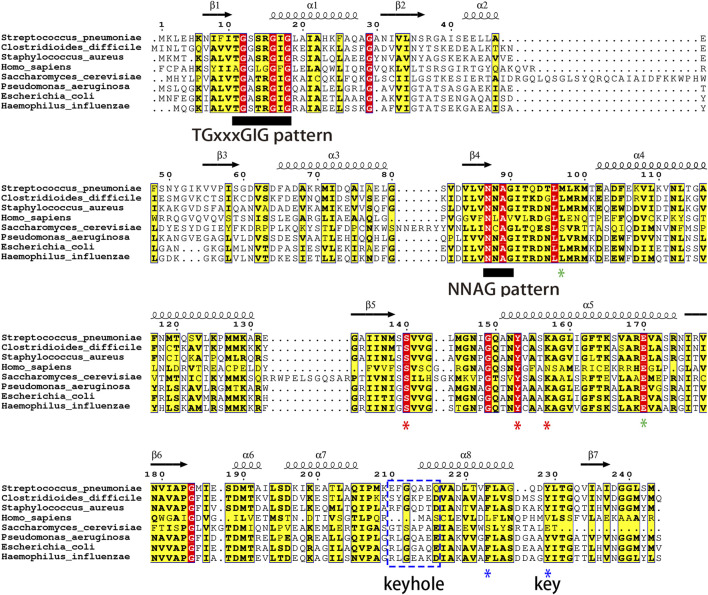
The Clustal MUSCLE alignment of 8 FabG species is visualized using ESPript. The positions of the active site SYK triad are marked with red asterisks. The conserved number 97 hydrophobic amino acids and Glu 170 of tetramer interface B, identified by structure-based alignment, are marked with green asterisks; The lock-and-key pattern and F/Y223 of tetramer interface A are indicated by blue asterisks and lines. Known functional motifs related to FabG are identified by black underlines, including the cofactor-binding motifs TGxxxGIG and NNAG.

Careful comparison of multiple structures of homologous proteins and the thorough analysis of large multiple sequence alignments is instrumental in identifying conserved patterns in sequence and structure, as well as highlighting critical interactions necessary for protein stability and function (Grant et al., 2007). Through the application of multiple sequence alignment to compare 8 proteins identified as FabG, it was observed that both the NADPH binding site and the catalytic triad have been conserved throughout evolution to maintain functioning FAS II systems, with the exception of human FASN, which exhibits considerable deviation. Notably, the human FabG domain within the FAS superstructure (KR) contains specific amino acid motif, including N-terminal cofactor binding sequence Glycine motif [TGXXXGIG] and NNAG motif (Oppermann et al., 2003; Kavanagh et al., 2008; Fujimoto et al., 2001). and the catalytic residue Lys157 is substituted by an Asn157 in the *H. sapiens* FASN variant. (Figure 1). Zhang et al. found that the activity of FabG is impacted by the key residue R15 (Zhang et al., 2013), which is also conserved in SpFabG but not in the human homologue. The conserved nature of S14, R15 and S36 but not D36 suggests that the enzyme utilize NADPH rather than NADH as substrates (Javidpour et al., 2014), further verifies the functioning of the SpFabG. In order to develop antibiotics targeting *Streptococcus pneumoniae* without affecting the host, the developed drug needs to have high specificity for SpFabG. Pairwise sequence alignments of SpFabG and human FASN suggested that they share only 22.3% identity, because of the three-dimensional structure depends on amino acid sequences, and it is reasonable to assume that there are sufficiently structural differences between SpFabG and human homologue. Additionally, after obtaining the structure of SpFabG (as detailed later), overlaying the predicted FASN alphafold model with the structure of SpFabG revealed noticeable differences, with an RMSD of 2.7 Å (data not shown), especially at the two interfaces where SpFabG forms the tetramer. Hence, there is specificity in targeting allosteric inhibitors against the tetramer interface of *Streptococcus pneumoniae* FabG.

The evolutionary divergence of gram-positive bacteria, gram-negative bacteria, and eukaryotes is well characterized by the evolutionary tree analysis (Supplementary Figure S3). The highest sequence identity match to SpFabG was observed in gram-negative bacteria, with *C. difficile* FabG showing a 68.7% resemblance and *S. aureus* FabG demonstrating a 66.1% similarity. Conversely, the most distant sequence similarity relation to SpFabG was found in *S. cerevisiae* OAR1 and the KR domain of the *H. sapiens* FASN super-enzyme, the human FabG equivalent.

### Structure features of SpFabG

**FIGURE 2:**
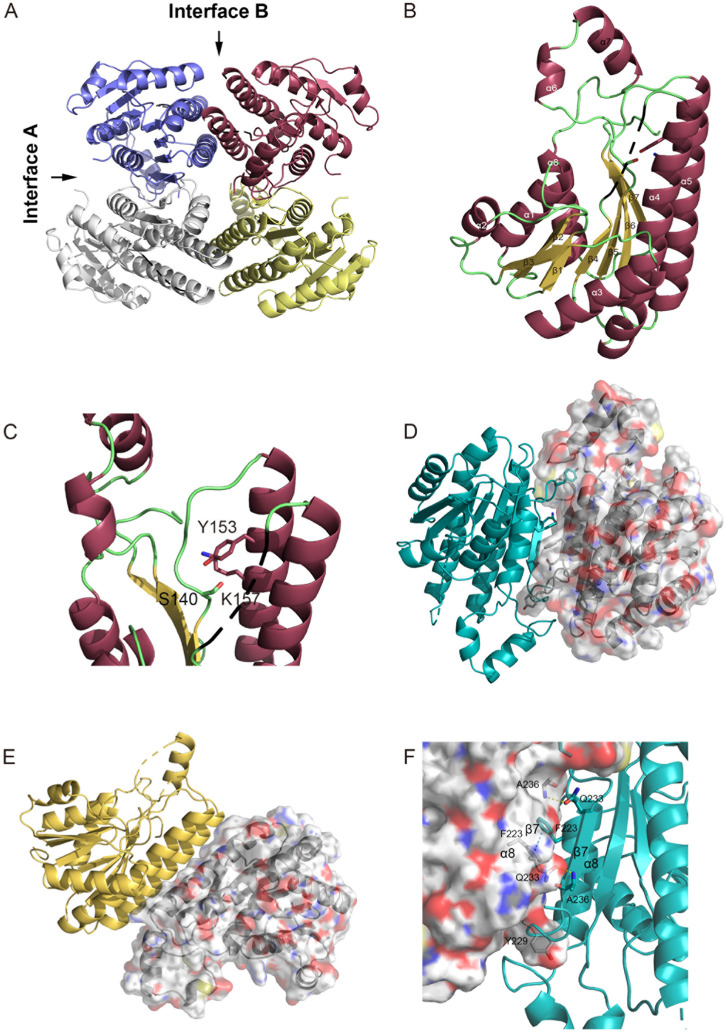
Structure of *S. pneumoniae* FabG. **(A)** The FabG tetramer is depicted with monomers shown in alternating color schemes for clarity. **(B)** A cartoon diagram of the monomer illustrates the secondary structural elements with their conventional numbering, and indicates the disordered loop of residues 92–98 by a black dash line and the active-site triad (Ser140, Tyr153, and Arg157) are shown as sticks. **(C)** Close up view of the active triad. The figure was made with PyMol. **(D–F)** Intermolecular interactions in S. pneumoniae FabG. Monomers displayed in different color schemes for visual clarity. The protein surface map is color-coded based on the residue charge: white for neutral, red for negative, blue for positive. Yellow for cysteine. The perspective view of two interacting monomers on interface A **(D)** and interface B **(E)**, to maintain clarity, other interfering monomers have been removed. Residues involved in polar interactions, π-π interactions and Tyr229 are represented by sticks. Detailed interactions of interface A are shown in **(F)**, hydrogen bonds by yellow dashed lines and π-π stacking by blue dashed lines. The figure was made by PyMol.

Despite research confirming FabG as an essential gene in *Streptococcus pneumoniae*, impacting its adaptability and morphology (Thanassi et al., 2002), there is currently a lack of reports on the structure of FabG. Revealing the protein structure of FabG is crucial for developing antibiotics targeting *Streptococcus pneumoniae*. The profound influence of AlphaFold (Jumper et al., 2021) on the field of structural biology has opened up opportunities for a growing cohort of scientists from non-structural biology backgrounds to explore and contribute to diverse fields, including drug discovery. We have determined the crystal structure of SpFabG at 2 Å resolution using AlphaFold predicted monomer as template for molecular replacement (Figure 2A), shows casing its similarity to the overall fold of the *E. coli* FabG (Price et al., 2001). AlphaFold monomer model was observed to align well with the crystal structure overall (Supplementary Figure S4A, RMSD = 1.9 Å) and the disparities are concentrated around functional loops near the NADPH binding site and interface B involved in dimer-dimer interactions to form tetramers. By aligning the structure of AlphaFold tetramer and crystal structure, we observed that AlphaFold accurately predicted interface A but exhibited errors in predicting interface B (Supplementary Figure S4B–D). This suggests that interface A likely harbors more conservative and stable interactions compared to interface B, potentially serving as a critical site influencing enzyme activity. Furthermore, in alignment with the findings from Wang (Wang et al., 2023) et al., that directly applying the predicted model for downstream structure-based virtual screening may not be suitable. In the asymmetric unit of the crystal form, there is a single tetramer. Each of the four monomers in the asymmetric unit adopts a similar fold, albeit with minor differences attributable to crystal contacts. Notably, the α6/α7 subdomain and the β4-α4 loop exhibit substantially higher B factors compared to the rest of the protein, indicating their flexibility and potential involvement in critical physiological functions. Despite the slight differences in the flexible region among the monomers, this analysis primarily focuses on the structure of the most stable monomer, chain A. Overall, the protein conforms to a Rossmann fold, with its core comprising a twisted, parallel β sheet consisting of seven β strands flanked on both sides by a total of eight α helices. The central structure is composed of two right-handed βαβαβ motifs, with helix α3 connecting the two motifs. The first motif is composed of strands β1, β2, and β3, and helices α1 and α2, while the second motif consists of strands β4, β5, and β6, and helices α4 and α5. The β5-α5 loop region retains flexibility, but in all active FabG-NADPH complexes, it could form a helix that extends away from the binding pocket upon cofactor binding. The α6/α7 flexible lid, located outside the active pocket, is distinct from the body of the protein. The final two secondary structure elements, α8 and β7, rejoin the protein’s body adjacent to strand β6. The active-site residues (Ser140, Tyr153, and Lys157) are clustered near the loop connecting β5 and α5 (Figures 2B, C). Notably, both Tyr 153 and Lys 157 reside on helix α5, while Ser140 is situated in the loop region at the carboxy-terminal end of β5, potentially forming a helix induced by NADPH binding. The orientation of the active triad directs away from each other in many apo-FabG homologues. The conformation observed in the crystal structure is considered to be inactive conformation, with the Ser140-containing β5-α5 loop occupying the pocket where the nicotinamide part of the ligand should be (Hou et al., 2016; Cohen-Gonsaud et al., 2002). In three out of the four monomers in the asymmetric unit, this loop is stabilized by a crystal contact, and the electron density clearly reveals both the conformation of the loop and the active triad. For the remaining monomers, without crystal contacts to stabilize the loop, the electron density, while weaker, distinctly depicts the same orientation of the active triad and loop positioning.

The SpFabG protein forms a tetramer through two distinct interfaces, detailed interactions are shown in Figures 2D–F. The interface A is established between the α8 helix and the β7 strand from each monomer. The two β7 strands run antiparallel, with specific side chain interactions bridging the gap between them. Phe223 in the α8 interface plays a crucial role by facilitating a π- π stacking and attracting the relevant side chains towards each other, and Ala236 in the β7 interface interacts with Gln233 in β7′ by forming a hydrogen bond. It is noteworthy that the hydrophobic side chain of Tyr229 inserts into a hydrophobic pocket on the neighboring side, resembling a key inserting a lock. The interface B, locates between helices α4, α5 and their respective counterparts in the neighboring monomer (α4′, α5′), constitutes a four-helix bundle. In this interface, α5 primarily interacts with α5′ and α4 interacts with α4′, indicating a same pattern observed in most FabG homologues. The interaction between α5 and α5′ is largely hydrophobic and involves several hydrophilic residues.

### Function of conserved pattern of interfaces and active triad

FabG is highly conserved responsible for fatty acid synthesis in prokaryotic and eukaryotic mitochondria (Shanbhag, 2019b; Vella et al., 2021; Hu et al., 2021). Despite its long research history, a consensus on its detailed catalytic mechanism has not been reached. Price et al. first proposed the active triad model of FabG, arguing that electron transfer in the redox reaction, which is catalyzed by FabG, must depend on three highly conserved amino acids known as SYK triad (Price et al., 2001; Price et al., 2004). Interestingly, FabG homologous protein in human FASN has a special case of K to N substitution and still has FabG function. Furthermore, researchers have recently reported that the SYK triad mutant protein of *E. coli* FabG is also functional enzyme (Hu et al., 2021), suggesting FabG homologues may possess similar but distinct catalytic mechanisms. While most FabG crystal structures are as homotetramer, certain FabG proteins exist primarily as homodimers in solution, such as *M. tuberculosis* MabA (Cohen-Gonsaud et al., 2002), Plasmodium falciparum (Wickramasinghe et al., 2006) and *Vibrio cholerae* FabG (Hou et al., 2016). FabGs from various organisms show variations in their solution oligomerization states, despite sharing tetrameric quaternary structures with similar dimer interfaces (Hou et al., 2012). The buried surface areas within dimer interface A exhibit consistency across FabGs, calculated at about 1,600 Å2 by PDBePISA (Krissinel and Henrick, 2007). However, it appears that differences in the stability of dimer interface B play a crucial role in the diversity of oligomerization states among FabGs in solution (Hou et al., 2016). To investigate the state of SpFabG in solution, analytical size-exclusion chromatography was conducted using 23 kDa and 100 kDa standard protein references. The molecular weight of SpFabG monomer is approximately 25.7 kDa, with dimer and tetramer forms at around 51.4 kDa and 102.8 kDa, respectively. Size-exclusion chromatography separates substances based on molecular weight, thus the elution order observed was tetrameric FabG, 100 kDa standard, dimeric FabG, monomeric FabG and 23 kDa standard, which allows us to determine the form of FabG based on its retention time relative to the standard proteins. The results indicate that apo-SpFabG predominantly exists in tetrameric form, but in the presence of the cofactor NADPH, it primarily exists as dimers (Supplementary Figure S6A), which is the first observation of a tetrameric quaternary structural change induced by the coenzyme NADPH in the FabG homologous protein. Thus, the dimer, rather than the tetramer, is likely the active conformation of the SpFabG. The calculations using PDBePISA indicate that the tetrameric crystal structure of SpFabG has a buried surface area of approximately 1,500 Å2 for interface A and 800 Å2 for interface B. This suggests that, similar to most FabGs, the larger interface A is likely more stable and serves as the interface responsible for maintaining the dimerization status in both tetramers and dimers.

**FIGURE 3:**
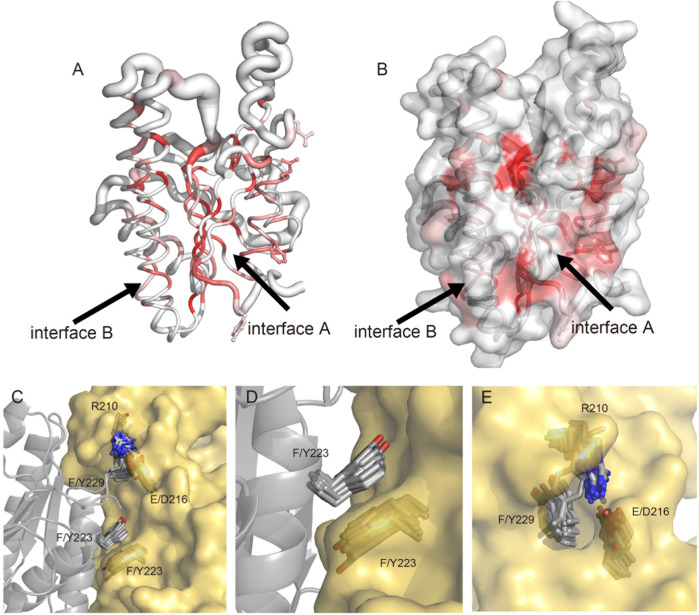
Visualization and superposition of sequence and structural conservation of FabG homologous proteins. **(A)** The sausage representation showing a tube depiction of FabGs, whose radius is proportional to the mean rms deviation per residue between Cα pairs. The tube is colored according to the level of sequence conservation, from white (low score) to red (identity). The conserved F223, Y229, E210 and Q216 are presented in ball-and-stick. **(B)** The surface representation of FabGs by its solvent accessible surface with the same color ramping and orientation as in panel **(A)**. Structure was made by ENDscript and visualized by PyMol. **(C–E)** The superposition of FabG structures from 37 species in the PDB database with S. pneumoniae FabG reveals a conserved tetramerization pattern. **(C)** The superposition of two monomers involved in the tetrameric interface A shows conserved residues R210, E/D216, F/Y223 and F/Y229. The two monomers of SpFabG are depicted in cartoon and surface representations, with the mentioned residues highlighted in stick form. Panel **(D)** illustrates the formation of a π-π interaction between conserved residues F/Y223. **(E)** The conserved lock-key pattern shows F/Y229 protruding into a hydrophobic pocket formed by R210 to E/D216.

Given the significant impact of the formation of the tetramer of *E. coli* FabG on enzyme activity (Lai and Cronan, 2004), as well as the development of inhibitors by disrupting the interaction of pathogenic microbial FabG tetramer interface residues (Cukier et al., 2013; Vella et al., 2021), we hypothesized that the interactions among monomers, particularly involving key amino acids at the tetramer interface, can potentially modulate enzyme activity allosterically. Consequently, factors capable of altering the FabG oligomeric state (e.g., amino acid mutations or inhibitor binding at the interface) may affect enzyme function. To validate this hypothesis, we conducted a detailed analysis of the essential interacting sites at the protein-protein interface and compared the conservation of these sites in the multiple sequence alignment with the structural comparison of other homologous proteins using ENDscript (Robert and Gouet, 2014) (Figures 3A, B). All homologous protein structures are superposed onto the SpFabG and the size of the tube is proportional to the root-mean-square deviation per residue between Cα pairs, representing the structural conservation. The white to red color ramping is used to visualize sequence conservation. The most conserved region in the sequence is found in the β-sheets of the protein core, shaping the NADPH substrate binding site. In contrast, the residues on the protein surface are relatively non-conserved. Notably, the structures of the two interfaces involved in homotetramer formation are structure-conserved, indicating that homotetramerization is a characteristic feature of FabG. Among the two interfaces, the residues of interface A are more conserved, confirming that interface A is the more stable one. Additionally, regions such as the β4-α4 loop, β5-α5 loop, flexible lid, and α2-β3-α3 show both non-conserved in sequence and structure.

By detailed aligning the structures of SpFabG and 37 reported FabG proteins from the PDB database (Supplementary Table S3), we found two sets of conserved patterns at interface A. Firstly, the conserved Phe/Tyr223 of one monomer interacts at the interface A through π-π stacking with neighboring Phe/Tyr223 (Figures 3C, D), with the exception of *S. cerevisiae* OAR1 (4FDA) and *Bacteroides* thetaiotaomicron FabG (3NYW). Secondly, the conserved nature of Phe/Tyr229, along with the spatially adjacent Arg210, hydrophobic residue211, Gly212, and Glu/Asp216 pattern in neighboring subunit, resembling a lock-and-key interaction, the hydrophobic side chain of Phe/Tyr229 extends outward from the middle of the α7-β7 loop, resembling a key inserts into the hydrophobic pocket, with certain amino acids showing preferences, for example, residue 210 tends to be arginine with long side chains, residue 211 tends to be a hydrophobic residue participating in forming and stabilizing the hydrophobic core of the flexible lid region, conserved Gly212 compromises its side chain to accommodate side chain of Phe/Tyr229, and Glu/Asp216 provides a long hydrophobic side chain to increase the depth of the keyhole. The gap located in lock-and-key pattern between the two subunits is sealed by the conserved R210 to E/D216 constituting the hydrophobic pocket, which is enhanced through the formation of hydrogen bonds and ionic interactions between long side chains in many structures (Figures 3C, E). The hydrophobic pocket is not highly sequence-conserved, but structure-conserved, which means R210 and E/D216 are consistently replaced together. The replaced residues still maintain long hydrophobic side chains, and the side chain heads tend to form polar hydrogen bonding interactions to create a hydrophobic and enclosed pocket. Additionally, we presumed that Phe/Tyr229 may be attracted into the hydrophobic pocket by the π-cation interaction with R210, and once fully inserted, the π-cation interaction weakens while the hydrophobic effect dominates to stabilize the tetramerization.

We also observed certain atypical FabG variants that display inconsistency with the conserved pattern of most previously reported FabG at interface A. In the FabG4 of *M. tuberculosis* (3V1T) (Supplementary Figure S5A), the N-terminus contains an additional “flavodoxin type” domain (Cheng et al., 2012b) compared to SpFabG, forms a dimer-like structure with rest of protein, closing the imaginary interface A to the protein’s interior region. This could be a result of a gene fusion event in the *Mycobacterium* genus, as the high molecular weight FabG4 is found in many *Mycobacterium* species. Therefore, despite the substitution of F/Y229 with A438, the dimer-like FabG4 structure is retained through embedding the interface A inside the protein. In the FabG of *Bacteroides* thetaiotaomicron (3NYW) (Supplementary Figure S5B), the F/Y223 is replaced by C218, maintaining hydrophobic attraction with the opposite C218. The substitution of F/Y229 with C227 and the loss of hydrophobic pocket led to the diminish of the hydrophobic attraction, yet many hydrogen bonds are formed in this region. It is possible that the attraction between C218 and the lock-and-key pattern is still at a substantial level, maintaining the dimerization. In the FabG of Aeropyrum pernix (5B1Y) (Supplementary Figure S5C), the F/Y229 is replaced by G236. The multiple sequence alignment results show a deletion of F/Y229 in the OAR1 of *S. cerevisiae* (4FDA), and the conserved F/Y223 is replaced by the hydrophilic S269 but still retains the hydrophobic pocket (Supplementary Figure S5C). Interestingly, both proteins lost the ability to form dimers at interface A, as observed in the crystal structure where monomers can only form homodimers through interface B as the biological unit. Based on the novel discovery of the conservative patterns at the FabG tetramer interface A, we inferred that the function of FabG relies on oligomerization, while the yeast OAR1 and Aeropyrum pernix FabG might have lost the ability to form tetramers through interface A during evolution but gained benefits from other variations, thus retaining these distinct homodimeric FabG enzymes. Cukier et al. were among the first to investigate FabG allosteric inhibitors, who conducted a thorough screening of potential inhibitors in a small molecule compound library, specifically focused on the ability of these inhibitors to bind to interface B of the *Pseudomonas aeruginosa* FabG biological unit (Cukier et al., 2013). Interestingly, the researchers noted that due to the significant rotational changes to the monomer at interface B following inhibitor binding, the conventional method of virtual screening from the structure of the apo protein was not viable. More recently, investigations have expanded to include the identification of µm-level inhibitors targeting the FabG enzymes of *Acinetobacter* baumannii and *Salmonella Typhimurium* through small molecule screening (Vella et al., 2021). These findings suggest that the design of allosteric inhibitors for FabG in other organisms is indeed feasible, highlighting the potential for broader applications of this approach in antibiotic development efforts.

**FIGURE 4:**
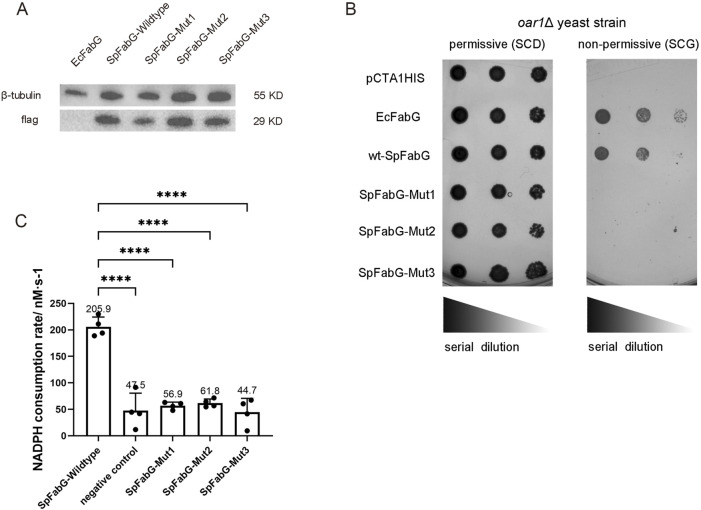
Identification of FabG activity *in vivo* and *in vitro*. **(A)** Western blot results indicated that both wild-type and mutant SpFabG were expressed in oar1-defective yeast cells. **(B)** A comparison of the growth status of a brewing yeast oar1 mutant strain was performed on two different culture media with differing carbon sources (fermentable carbon source SCD and non-fermentable carbon source SCG). The experiment included yeast strains carrying *Escherichia coli* FabG as a positive control, yeast strains carrying an empty vector pCTA1HIS as a negative control, and yeast strains carrying either wild-type or mutant FabG. These strains were cultured on SC-glucose plates at 30°C for 2 days or on 3% (w/v) SC-glycerol plates at 30°C for 5 days before capturing images. A series of 10-fold diluted bacterial suspensions were manually spotted on the plates. **(C)** The *in vitro* enzymatic activity of FabG was determined. The assay involved preparing the enzyme reaction mixture in a 96-well plate, consisting of 1 mM NADPH, 1 mM AcAcCOA, and 0.023 mg/mL of wild-type/mutant FabG. The average reaction rate of NADPH within the first 5 min was then measured. Remarkably different catalytic activities were observed between wild-type FabG and the three mutant forms of FabG. Statistical significance levels were defined as ^*^ *p* < 0.05, ^**^ *p* < 0.01, ^***^ *p* < 0.001, and ^****^ *p* < 0.0001.

To investigate the impact of the catalytic triad and conserved lock-and-key pattern on quaternary structure and enzyme function, three SpFabG mutants were constructed: mut1 (S140A Y153F K157A) destroyed the conserved catalytic triad, mut2 (K2D, F223D, Y229E, Q233G) and mut3 (K2D, F223D, Y229F, Q233G) destroyed the essential non-covalent amino acid interactions at the dimer interface A. Three mutants displayed a decreased proportion of tetramers and an increased proportion of dimers and monomers, suggests that all three mutants destabilize the FabG tetrameric structure to some extent and the addition of the NADPH cofactor led to the dimer becoming the predominant component, indicating that the three mutants retain the ability to bind NADPH (Supplementary Figure S6A–D). This observation is consistent with the wild-type SpFabG, where NADPH binding promotes dimer formation. To investigate whether three FabG mutants retain enzyme function, we constructed the FabG mutants into a yeast expression vector pCTA1HIS and then transformed them into the oar1Δ strain for *in vivo* functional experiments. The results revealed that all three SpFabG mutants, although present in yeast at similar protein levels, failed to restore the growth of yeast defective strains on selective media, unlike the wild-type SpFabG and *E. coli* FabG (Figures 4A, B). To further ascertain the inactivation effects of the mutations, the wild-type and mutant SpFabG proteins were produced and purified using *E. coli* expression system for *in vitro* enzyme assays. The results showed a significant difference in catalytic activity between the mutant and wild-type SpFabG, with no significant difference from the negative control (Figure 4C), indicating the critical importance of both the active site triad and dimer interface A sites for SpFabG function.

### Virtual screening and molecular dynamics simulations

Based on our understanding of the conservation of the FabG homologous protein at interface A and the essentiality of the SpFabG tetramerization site for enzymatic activity, we propose designing allosteric inhibitors targeting the conserved interface A. For molecular docking, the binding site pocket should be elaborately mapped to fit any suitable ligand into the cavity of the receptor and to develop a novel drug compound, one must first find a precursor compound that can potentially inhibit the receptor activity by binding to its active site or other ways with high affinity, which may cause kinetic inhibition of the receptor (Cerqueira et al., 2015). Both methods, CB-Dock2 and DoGSiteScorer, predicted a common SpFabG binding pocket and DoGSiteScorer is preferable with a “drug score” of 0.49, therefore more likely to be an authentic drug-targetable region, interestingly, this pocket is exactly the conserved lock-and-key pattern. It is evident this particular sequence is not conserved among FabG homologues as shown in Figure 1, leading to diversity in the physicochemical properties of the pocket it forms. Particularly noteworthy is the deletion observed in human FabG homologues at this location, further enhancing the selectivity of the inhibitor towards SpFabG. Therefore, selecting this site as the target for inhibitor design not only confers bacterial species specificity but also significantly reduces the likelihood of adverse effects in humans.

We initiated our study by employing the virtual screening technique to identify potential inhibitors with adequate binding affinity. To achieve this, we started with a chemical database comprised of 533,600 small molecules, taking into account the hydrophobic nature of the binding site. After preparing the ligands, a pharmacophore model was generated using PHASE (Dixon et al., 2006), incorporating a total of 7 characteristics: two hydrogen bond donors, two aromatic rings, and three hydrogen bond accepters. Following this, a screening process retained compounds that met four or more characteristics, resulting in an initial pool of approximately 200,000 compounds. These molecules were then subjected to Glide (Friesner et al., 2006), which utilizes the concept of the shape of binding pockets and electrostatic potential resemblance to select new molecules may show similar binding modes to the binding pocket. Based on the docking scores, the binding free energy of the top 1% ligands were then calculated using Prime Package of Schrodinger. Finally, the top 5 compounds ranked by the binding free energies were identified as potential inhibitors for SpFabG and their physicochemical and pharmacokinetic ADMET parameters were detailed assessed via ADMETlab (Xiong et al., 2021) (Supplementary Table S2).

Detailed interaction patterns of protein pocket and ligands are shown in Supplementary Figure S8. Ligand 1 is the most stable compound ranked by binding free energy. Ligand 1 (ZINC000000451341) was found to form one hydrogen bond with Glu210, mimic the role of Tyr229 in a dimer interface, stabilize the hydrophilic end of the compound, and position its hydrophobic end in the preferred environment. Ligand 2 (ZINC000004494577) is special in that it does not contain rotatable bond, was shown to form two hydrogens bonds with Met185 and Glu210, while the rigid core is located within the hydrophobic pocket. Ligand 3 (ZINC000409436437) was not found to form any polar interaction with binding site as can be seen from the 2D interaction diagram, and the Tyr229 pocket was partially occupied, with the compound mainly binding to the hydrophobic plateau outside the pocket, which could be predicted that this binding mode was not stable enough. Ligand 4 (ZINC000001351262) imitated Tyr229 by a hydrophobic benzene ring, anchoring an oxygen atom to the binding site using three consecutive residues (Asp238, Gly239, Gly240). Ligand 5 (ZINC000075629401) was found to form two hydrogen bonds with Glu210 and Gly239, and the phenol part had the similar binding pattern with Tyr229.

A chemical cannot be a drug, no matter how active nor how specific its action, unless it is also taken appropriately into the body (absorption), distributed to the right parts of the body, metabolized in a way that does not instantly remove its activity, and eliminated in a suitable manner. A compound must get in, move about, hang around, and then get out. ADMETlab 2.0 (Xiong et al., 2021) successfully conducted the ADMET assessment, which identified specific properties relevant to the potential of the compounds being utilized as drugs. The ADMET values of all ligands fell within the appropriate ranges and satisfied the criteria defined by Lipinski’s ‘rule of five’, a measure utilized to evaluate the drug-likeness of a compound. Although the PPB (Plasma Protein Binding) or LogS parameter for L1, L2 or L4 deviates slightly from the optimal range, this discrepancy can be resolved by employing standard lead optimization procedures on the stronger binders, thus sacrificing less overall affinity.

Molecular dynamic simulations are computational technique used to study biomolecules in a virtual environment. By simulating the binding process between proteins and small molecules, valuable information on binding modes, kinetics, binding energy, affinity, and selectivity is revealed, which aids in predicting the strength and specificity of complex formations, facilitating the evaluation of drug molecules’ binding abilities with target proteins to guide drug design and optimization. Molecular dynamics simulations rely on unbroken protein-ligand complexes as the starting system. Due to the presence of unmodeled flexible regions in the SpFabG structure, a full-length protein structure needs to be constructed. The construction of the chimeric full-length SpFabG structure involved utilizing the SpFabG crystal structure we solved and the *S. aureus* FabG structure (PDB: 3OSU) as the homology modelling template, chosen due to its 50% sequence identity over a 97% sequence coverage. This approach was based on the recognition that the chosen modelling template, SaFabG, was termed the apo-FabG, implying that the resulting SpFabG model likely acquired an approximate apo-enzyme structure in concordance with the protein backbone. The homology modelling was carried out in MODELLER (Webb and Sali, 2021), a Python script-based homology modelling program designed to computationally model folds and energies of an amino acid sequence in alignment with the template structure (Supplementary Figure S7A). Following this, the monomeric full-length SpFabG model was validated, as outlined in the Methods, using various structure validation servers. These servers were utilized to compare the model with their curated databases of structure parameters derived from experimental structure modelling methods such as X-ray crystallography and NMR spectroscopy. All quality assessment servers found that the structure of the SpFabG model closely resembled experimentally determined protein models on a local protein fold basis, positioning the SpFabG model within an acceptable range (Supplementary Figure S6D–F). In addition, a detailed Ramachandran plot analysis of SpFabG torsion angles was performed using the SAVES server, revealing that 195 amino acids (90.7%) fell within the preferred region, 19 residues (8.8%) were situated in the “acceptable region,” with only one outlier. The SpFabG 3D model appears to contradict current physiochemical theories when evaluating the torsion/rotation angles of the α and β carbons within the peptide bonds. However, the findings indicate no serious covalent geometry flaws in the model. ProSA, a server used for evaluating protein model quality, placed the SpFabG homology model within an acceptable range, yielding a resulting z-score of −7.11. The server compares the energy score and residue energy fluctuations of the model with those of experimentally determined structures. The general residue scores for SpFabG also fall within the acceptable range. The z-score plot illustrates the correlation between computed z-scores and sequence length for experimentally discovered protein structures, with separate regions for structures determined by NMR and X-ray crystallography. The SpFabG 3D structure homology model is situated within a region of the graph where X-ray structures are concentrated. Additionally, the energy graph visualizes the different energy fluctuations in each residue by applying two separate window sizes.

**FIGURE 5:**
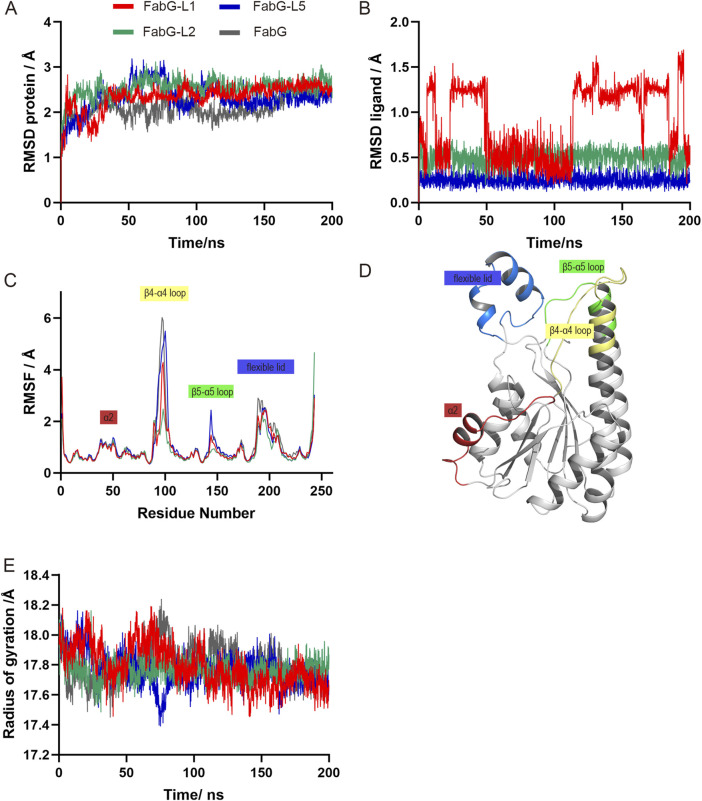
The MD simulation results of SpFabG-Lx and apo enzyme obtained from the Gromacs molecular dynamics simulation. RMSD (root mean square deviation) distributions of the proteins **(A)** and ligands **(B)** across 100 ns simulation. RMSF (root mean square fluctuation) trends of the structures during the simulation are depicted in panel **(C)**, with specific sub-structures highlighted to illustrate significant fluctuations. **(D)** Three-dimensional visualization of moving region on SpFabG throughout the course of the simulations. **(E)** Radius of gyration changes within 200-ns simulated time.

We performed MD simulation of the top 5 complexes to measure the stability of the protein–ligand complex, except FabG-L1, L2 and L5 complexes which are stable during 200 ns simulation, ligand dissociation occurs in other systems (data not shown). For clarity, only these stable complexes were further analyzed. RMSD (root mean square deviation) profiles of the protein or ligand are shown in Figures 5A, B. The RMSF (root mean square fluctuation) calculates the fluctuation of each atom relative to its average position, indicating the average structural changes over time and providing a measure of flexibility in different regions of the structure. The RMSF plot constructed with all studied SpFabG-Lx complexes and the unbound SpFabG allowed for the clear distinction of all potential dynamic regions on the protein. Relevant regions on the 3D protein structures were color-coded according to the residue order, with α2 (red), β4-α4 loop (yellow), β5-α5 loop (green) and flexible lid (blue) being delineated (Figures 5C, D). In the 200 ns simulation, the apo protein and three complexes maintained stable with RMSD values below 3 Å. During the simulation of the SpFabG-L1 complex, it was observed that L1 initially caused an increase in RMSD values for the ligand at 6.1 ns, followed by a conformational change resulting in a displacement of up to 1.2 Å. In the SpFabG-L2 complex, both the ligand and protein showed high stability, with ligands showing an average RMSD value below 0.5 Å. The fluctuations in the four most dynamic regions of SpFabG have noticeably decreased, indicating a high affinity between L2 and protein binding, which suppresses the movement of flexible regions. In the case of the SpFabG-L5 system, both the protein and ligand maintained consistently lower RMSD values, and remained in their initial positions throughout the simulation, while the binding of L5 does not reduce protein fluctuations and the active site Ser140 containing β5-α5 loop exhibited increased dynamics. All suggested that the compound forming the SpFabG-L2 complex established the most stable interaction with SpFabG during the entire simulation. The radius of gyration graphs for the atomic coordinates of the apoenzyme and SpFabG-Lx complexes were also analyzed. During the 200 ns simulation, it was observed that the compactness of the SpFabG-Lx exhibited similar patterns of radius of gyration compared to the apo-enzyme, as depicted in Figure 5E.

The hydrogen bonds formation was examined using VMD hydrogen bond tools (data not shown) and visualized using Maestro (Supplementary Figure S9). In the SpFabG-L1 complex, the O atom of Asp238 maintained a hydrogen bond with the O4 atom of L1 for 75% of the time. The N atom of Met185 formed a hydrogen bond with the O4 atom of the ligand for 64% of the time. Additionally, the N atom of Gly239 established a hydrogen bond with the O2 atom of L1 for 46% of the time. These longer-lasting hydrogen bond interactions highlight key structural features of the interactions within the complex. The SpFabG-L2 complex demonstrates fairly stability, a consistent hydrogen bond between the N5 atom of L2 and the O atom of Glu210 maintained for over 88% of the time. Secondly, the O atom of Met185 formed a hydrogen bond with the O2 atom of the ligand for 31% of the time. In the SpFabG-L5 complex, the stability of the two sets of hydrogen bonds is remarkable. The hydrogen bond between the O2 atom of L5 and the O atom of Glu210 is consistently maintained for over 98% of the simulation time. Similarly, the hydrogen bond between the O1 atom of the ligand and the N atom of Gly239 is formed for more than 93% of the simulation time. These strong hydrogen bonds served to firmly anchor the two aromatic rings of the ligand within the binding pocket. Consequently, the RMSD of these three ligands in the complex system remains consistently at an exceedingly low value, further confirms that these complexes exhibited a stronger binding affinity among the hits.

We utilized VMD’s Timeline analysis tool to examine the secondary structure alterations in both the apoenzyme and ligand-bound SpFabG conformations as shown in Supplementary Figure S10. The most common and significant reconfiguration change, found in all three complexes, occurs at the NADPH binding site in the β4-α4 loop and in the N and C-terminal loops of the flexible lid, were already identified as highly flexible regions of SpFabG and had an impact on enzyme functionality. Specifically, the most significant changes of the L1 complex involved several key structural alterations. Firstly, the β3-α3 loop region in the SpFabG-L1 is more stable compared to that of apo-SpFabG, characterized by a decrease in coil and an increase in β-turn. There was a stabilization in the β4-α4 loop, where Ser140 is located, appeared a persistent β-bridge. Simultaneously, the active triad SYK containing β5-α5 loop underwent a transformation from coil to a long-term β-bridge. The β6-α6 loop outside the active pocket experienced a β-turn to β-bridge transformation, that was the same in all three complexed. The α7-α8 loop, participated in the interface B formation, maintained a β-bridge rather than coil structure almost the entire time and was seen in rest of the complexes. All of these changes enhanced the stability of the protein’s monomeric structure and reinforced the integrity of internal arrangement. The conformation changes observed in the L2 or L5 complex shared similarities but distinctions with the alteration seen in the L1 complex. In SpFabG-L2, there was a stabilization of coil to β-turn transformation in β3-α3 loop and the β4-α4 loop transformed from a flexible structure in the apo-FabG state to a dominant β-turn conformation when binding it. In SpFabG-L5, the most dynamic region, the β4-α4 loop, did not exhibit significant stabilization with the addition of L5, as can also be inferred from the RMSF plot (Figure 5C).

Combining all the structural analysis, we identified L1, L2 and L5 as a promising candidate for SpFabG inhibitor, based on the molecular docking scores obtained in this study and the observation that these ligands exhibited the highest affinity to SpFabG, as well as a more stable binding mode in comparison to other evaluated inhibitors.

## Conclusion

*Streptococcus pneumoniae* is a leading bacterial cause of various infections such as pneumonia, otitis media, and meningitis, but it has become resistant to multiple antibiotics, resulting in treatment failures, increased morbidity, and mortality rates. In prokaryotic cells including *S. pneumoniae*, fatty acid synthesis is essential for the cellular material composition, energy metabolism and biofilm formation. FabG, a single-form enzyme, is responsible for vital *de novo* fatty acid synthesis and is essential for S. pneumoniae. When SpFabG amino acid sequence was investigated through multiple sequence alignment, there is a conserved NADPH binding site along with the Ser-Tyr-Lys active triad responsible for substrate catalyzation. The functionality of this enzyme was verified by *in vivo* and *in vitro* assays. The 3D structure of SpFabG was achieve by X-ray diffraction method and further reconstructed by homology modeling to fill the experimental uncertain flexible region. Through mutational experiments, we have confirmed the hypothesis that the mutation of the conserved tetramerization site, as previously observed in *E. coli* FabG, leads to a loss of enzymatic activity, highlighting its crucial role in protein oligomerization and function. Building on this discovery, we performed virtual screening and molecular dynamics simulations to identify the 5 most optimal ligand compounds from a vast library containing 533,600 small drug-like compounds. These compounds were deemed suitable for lead optimization, and were subjected to binding free energy calculation using the MM/PBSA method. Subsequently, an investigation of their ADMET parameters was conducted, and were further evaluated through a 200 ns standard molecular dynamics simulation. The molecular dynamics (MD) simulation data revealed that the Rossman Fold structure, present in most beta-ketoacyl reductase (BKR) enzymes including SpFabG, expanded to form the tightest bonded complex. It was shown that the most flexible sites of SpFabG were the β4-α4 loop, β5-α5 loop, and flexible lid. The highest affinity SpFabG binders were identified as L1, L2 and L5, based on the binding free energy calculation and standard MD analysis. These compounds emerged as the most desirable lead compound for drug design against a single-form enzymatic step of a vital fatty acid pathway specific to *Streptococcus pneumoniae*.

## Data Availability

Publicly available datasets were analyzed in this study. This data can be found here: Streptococcus pneumoniae FabG (UniProt accession Q8DR15), Clostridioides difficile FabG (Q18B45), Staphylococcus aureus FabG (Q6G9Y2), Pseudomonas aeruginosa FabG (O54438), Haemophilus influenzae FabG (P43713), Escherichia coli FabG (P0AEK2), Saccharomyces cerevisiae FabG (P35731) and Homo sapiens FAS (P49327). The X-ray crystallographic coordinates and structure factor files for SpFabG structures are available in the Protein Data Bank (http://www.rcsb.org) under accession number 8X89. All data generated or analyzed during this study can be found in this published article and are also available from the corresponding author upon reasonable request.
