# Supplementary material for: Structure features of Streptococcus pneumoniae FabG and virtual screening of allosteric inhibitors
Source: Front Mol Biosci. 2024 Sep 27;11:1472252. doi: 10.3389/fmolb.2024.1472252 (PMC11467476; doi:10.3389/fmolb.2024.1472252)
Supplement: Supplementary file 1 [file Table1.DOCX]

## Supplementary Table

Table S1 Primary screening of protein crystallization conditions.

| Condition | Components |
| --- | --- |
| PEGION E11 | 0.1 M Sodium malonate pH 4.0, 12% (w/v) Polyethylene glycol 3,350 |
| JCSGII D7 | 0.2 M Sodium chloride 0.1 M cacodylate pH 6.5 20% PEG 1000 |
| JCSGII E2 | 0.1 M MES pH 6.0, 10% (w/v) (±)-2-Methyl-2,4-Pentanediol |
| JCSGII E5 | 0.1 M MES pH 6.0, 5% (v/v) PEG 6000 |
| JCSGII F4 | 0.1 M Sodium chloride 0.1 M Acetate pH 4.5, 30% (v/v) PEG-200 |
| JCSGII F5 | 0.1 M Acetate pH 4.5, 40% (v/v) 1,2-propanediol |
| JCSGII F7 | 0.1 M Sodium Acetate trihydrate pH 5.0, 10% (w/v) (±)-2-Methyl-2,4-Pentanediol |

Table S2. ADME parameters of the top 5 spFabG ligand candidates discovered via docking.

|  | Ligand | MW | PSA | nRot | nHD | nHA | QPlogPo/w | LogS | PPB | hERG Blocker | HOA | Lipinski |
| --- | --- | --- | --- | --- | --- | --- | --- | --- | --- | --- | --- | --- |
| L1 | ZINC000000451341 | 244.07 | 76.494 | 4 | 2 | 4 | 1.676 | -3.285 | 96.47% | 0.029 | 3 | Accepted |
| L2 | ZINC000004494577 | 248.03 | 139.968 | 0 | 1 | 10 | -1.285 | -4.564 | 87.89% | 0.043 | 2 | Accepted |
| L3 | ZINC000409436437 | 234.11 | 71.38 | 4 | 0 | 6 | -1.483 | -0.3 | 13.14% | 0.026 | 2 | Accepted |
| L4 | ZINC000001351262 | 233.08 | 47.37 | 2 | 0 | 2 | 2.674 | -4.512 | 95.64% | 0.105 | 3 | Accepted |
| L5 | ZINC000075629401 | 237.08 | 47.487 | 1 | 1 | 3 | 2.345 | -3.133 | 89.72% | 0.035 | 3 | Accepted |

MW: molecular weight (130.0/725.0); PSA: Polar van der Waals surface area (7.0/200.0); nRot: The number of rotatable bonds (0/11); nHD: The number of hydrogen bond donors (0/7); nHA: The number of hydrogen bond acceptors (0/12); QPLogPo/w: The predicted octanol/water partition coefficient (-2.0/6.5); LogS: The estimated water solubility value (-4/0.5); PBB: Plasma Protein Binding (<90%); hERG Blocker: The probability of being hERG blocker; HOA: Human oral absorption (1 is low, 3 is high) predicted on a 1 to 3 scale; Lipinski : MW≤500, logP≤5, nHA≤10, nHD≤5, if two properties are out of range, a poor absorption or permeability is possible, one is acceptable.

Table S3 The FabG of 37 species reported in the PDB database.

| PDB id | Organism |
| --- | --- |
| 2P68 | *Aquifex aeolicus* VF5 |
| 2UVD | *Bacillus anthracis* str. Ames |
| 2C07 | *Plasmodium falciparum* 3D7 |
| 1Q7B | *Escherichia coli* |
| 3OP4 | *Vibrio cholerae* O1 biovar El Tor str. N16961 |
| 6T7M | *Salmonella enterica* subsp. enterica serovar Typhimurium str. LT2 |
| 3GRP | *Bartonella henselae* str. Houston-1 |
| 5CDY | *Yersinia pestis* |
| 4RZH | *Synechocystis sp*. PCC 6803 |
| 4M8S | *Neisseria meningitidis* FAM18 |
| 3FTP | *Burkholderia pseudomallei* |
| 3F9I | *Rickettsia prowazekii* |
| 5OVJ | *Mycolicibacterium smegmatis* MC2 155 |
| 7CAW | *Acinetobacter baumannii* |
| 3LYL | *Francisella tularensis* subsp. tularensis SCHU S4 |
| 4NBV | *Cupriavidus taiwanensis* |
| 7CZC | *Vibrio harveyi* |
| 1ULS | *Thermus thermophilus* |
| 4NBR | *Brucella abortus* bv. 1 str. 9-941 |
| 5X8H | *Chryseobacterium sp*. CA49 |
| 4J2H | *Sinorhizobium meliloti* 1021 |
| 7B81 | *Azotobacter vinelandii* DJ |
| 4FDA | *Saccharomyces cerevisiae* S288C |
| 6T77 | *Klebsiella pneumoniae* |
| 4JRO | *Listeria monocytogenes* EGD-e |
| 3OSU | *Staphylococcus aureus* subsp. aureus Mu50 |
| 7X5J | *Candidatus Methanoliparum* |
| 3V1T | *Mycobacterium tuberculosis* H37Rv |
| 5B1Y | *Aeropyrum pernix* K1 |
| 7EMG | *Serratia marcescens* |
| 3NYW | *Bacteroides thetaiotaomicron* |
| 6T6P | *Klebsiella pneumoniae* 30684/NJST258_2 |
| 4GLO | *Burkholderia multivorans* ATCC 17616 |
| 3ICC | *Bacillus anthracis* str. 'Ames Ancestor |
| 4CQM | *Homo sapiens* |
| 4DML | *Synechococcus elongatus* PCC 7942 = FACHB-805 |
| 4AFN | *Pseudomonas aeruginosa* PAO1 |
