# Supplementary material for: Structure features of Streptococcus pneumoniae FabG and virtual screening of allosteric inhibitors
Source: Front Mol Biosci. 2024 Sep 27;11:1472252. doi: 10.3389/fmolb.2024.1472252 (PMC11467476; doi:10.3389/fmolb.2024.1472252)
Supplement: Supplementary file 2 [file Image1.pdf]

Supplementary Figures

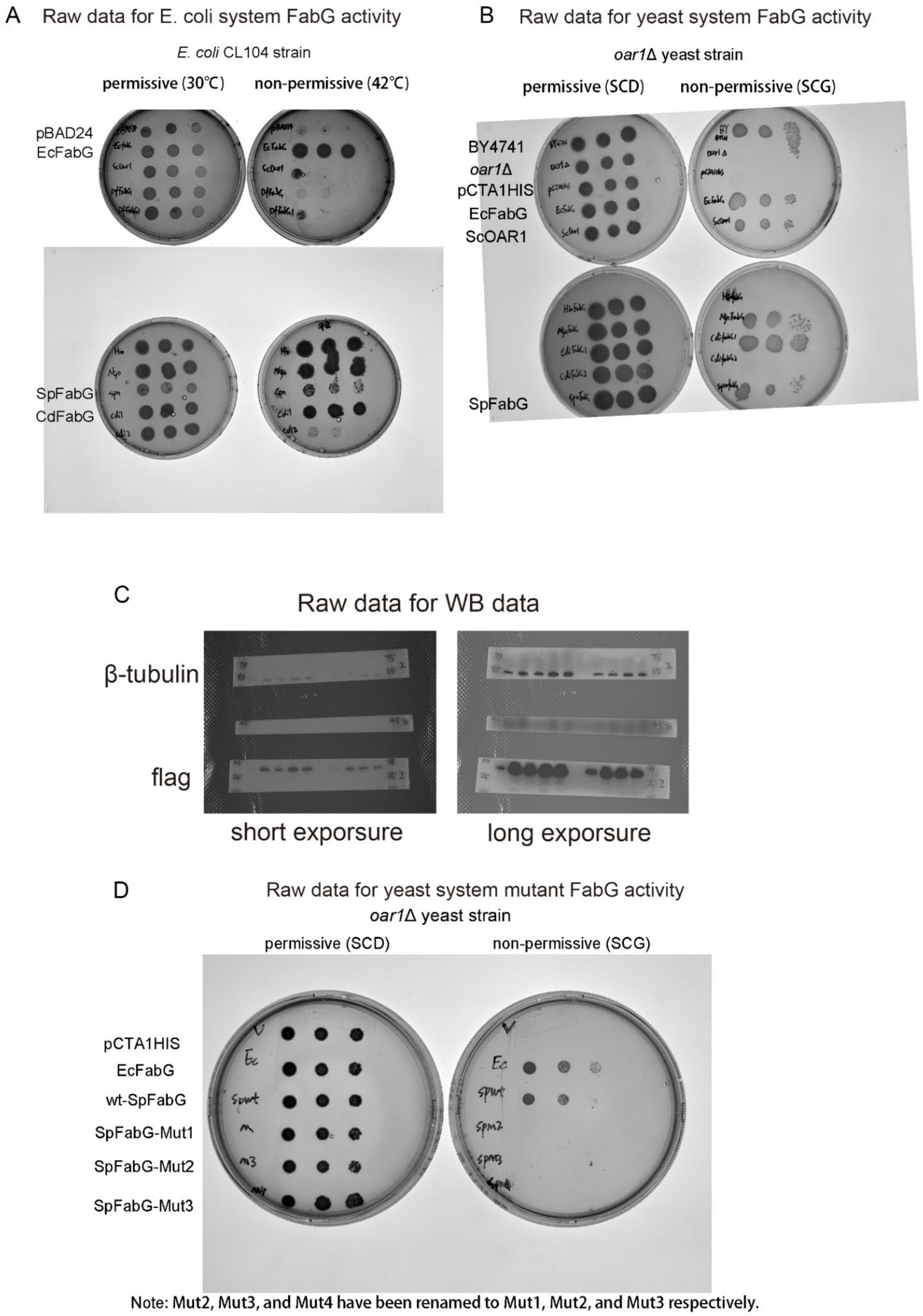

Figure S1. Raw data for *in vivo* FabG activity and WB data.

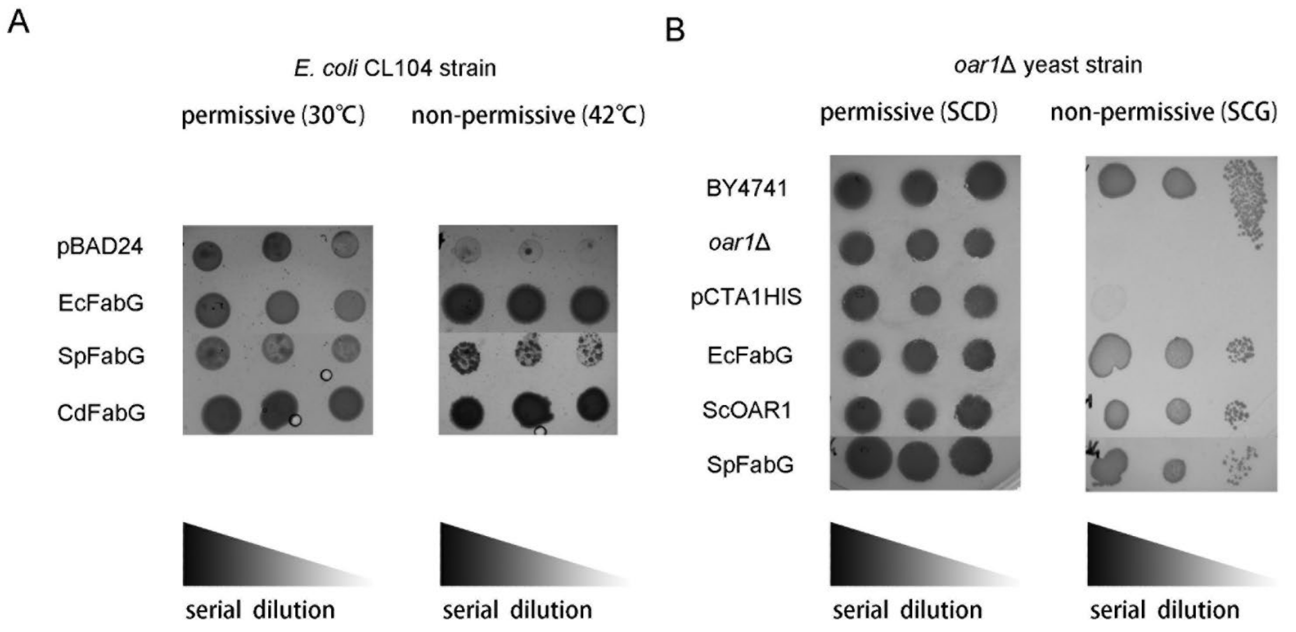

Figure S2. The defect phenotype was rescued by SpFabG. (A) The growth of *E. coli fabG* mutant strain CL104 was verified, transforming functional FabGs or empty vector, at both permissive and non-permissive temperatures. Functional FabGs including *E. coli* FabG, *Clostridium difficile* FabG. Strains were cultured on LB plate containing 0.1% (w/v) L-arabinose. (B) The growing ability of transformed *S. cerevisiae oar1*Δ strains (strain background: BY4741) was verified on two media: fermentable SC-glucose (SCD) and nonfermentable SC-glycerol (SCG). The experiment included the wild-type *S. cerevisiae* (positive control), *oar1*Δ and *oar1*Δ transformed with empty vector pCTA1HIS (negative control), and *oar1*Δ transformed with functional *E. coli* FabG and *Saccharomyces cerevisiae* OAR1 (positive control). The strains were cultured on SCD plates for 3 days at 30°C, or on SCG plates for 1 week at 30°C before photographs were taken. A series of 1:10 dilutions were spotted onto the plates.

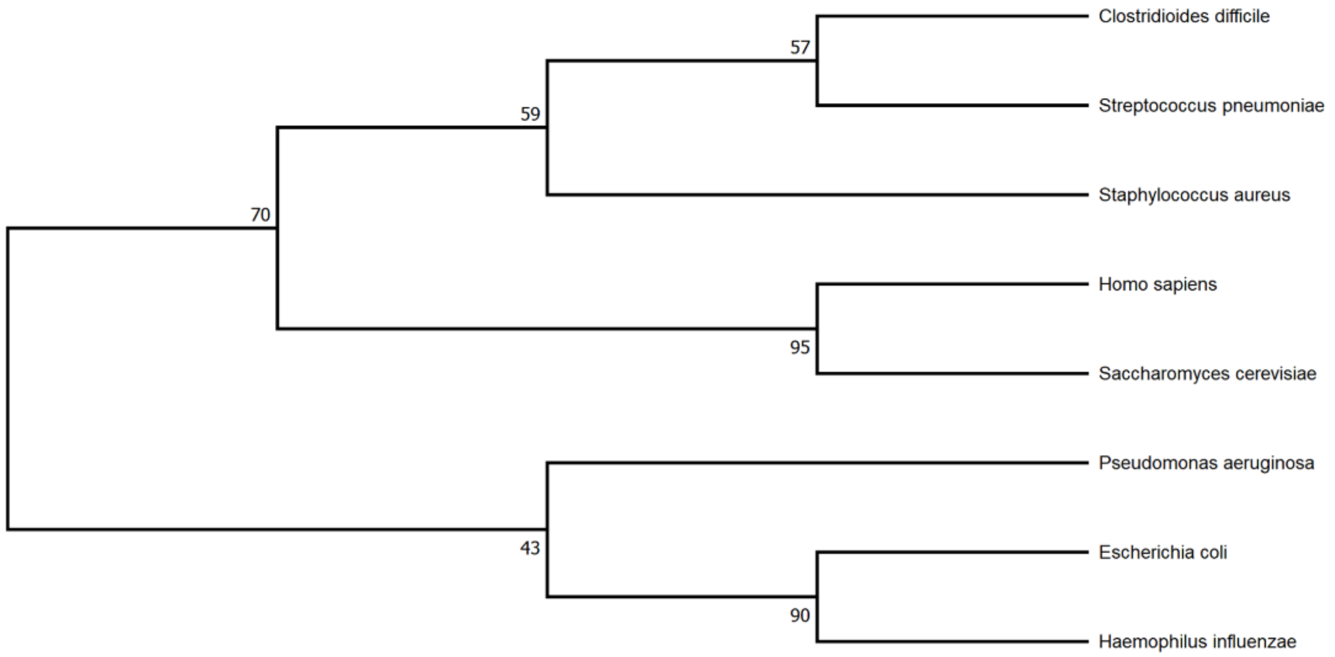

Figure S3. Phylogenetic and molecular evolutionary analysis of FabG from different organisms and several other related proteins were conducted using MEGA. Number above or below branches refers to the probability of each clade.

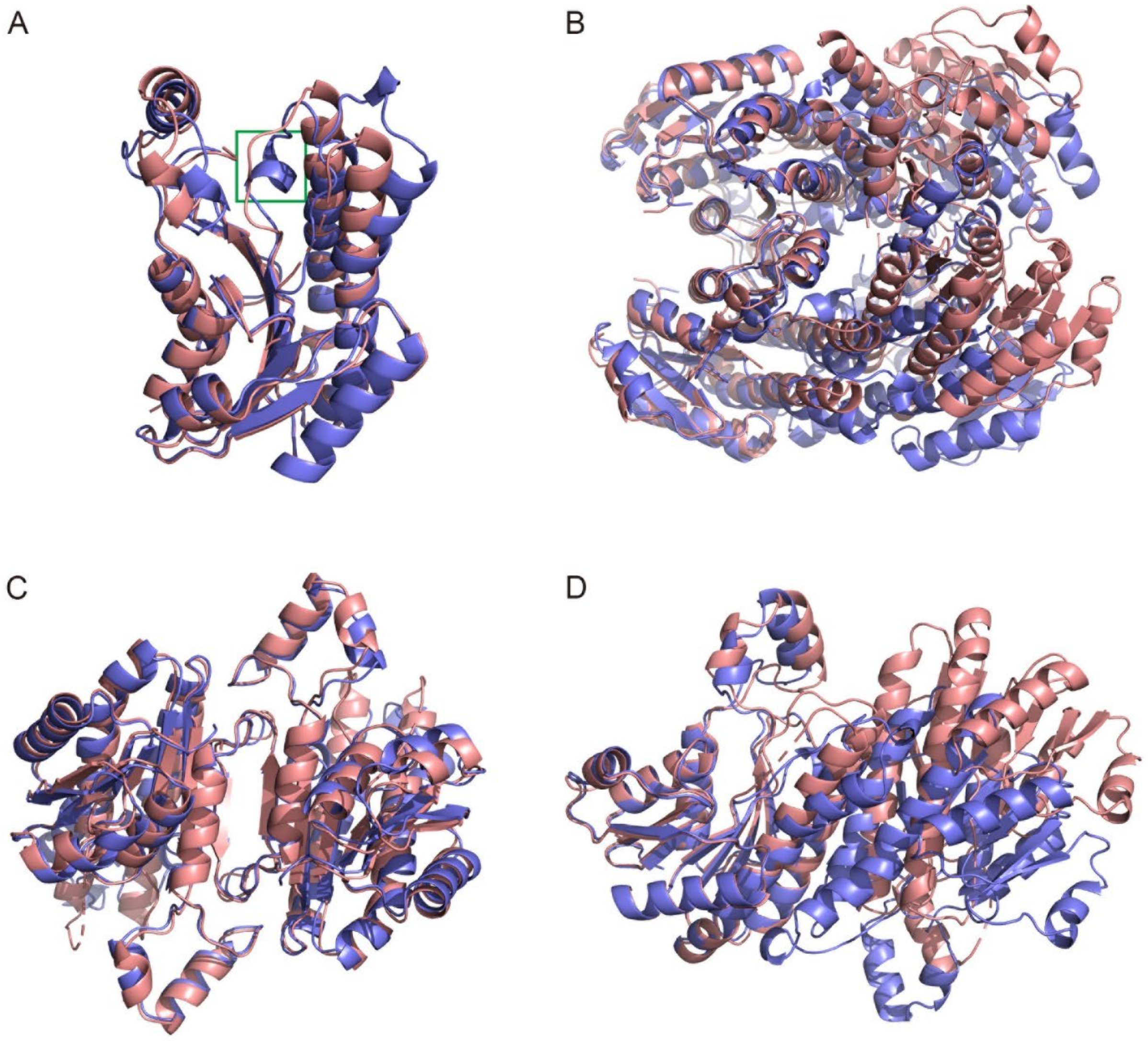

Figure S4. Overlay of the crystal structure of SpFabG with the AlphaFold model. (A) Overlay of MODELLER chimeric model (in red) with the predicted monomer (in blue). The RMSD between the protein main chain is 1.9 Å. AlphaFold prediction of multiple functional loops near the NADPH binding site is inaccurate, especially the β5-α5 loop highlighted in the green box, which it predicted as holo-conformation. (B) Overlay of the crystal structure (in red) with the predicted tetramer (in blue), showing significant differences (RMSD = 2.5 Å), particularly at interface B participating the dimer-dimer interaction. (C, D) Close-up views of interface A and interface B demonstrating that AlphaFold performs well in predicting the interactions at the conserved interface A (RMSD = 1.4 Å), but shows poorer performance in predicting interactions at interface B (RMSD = 3.7 Å).

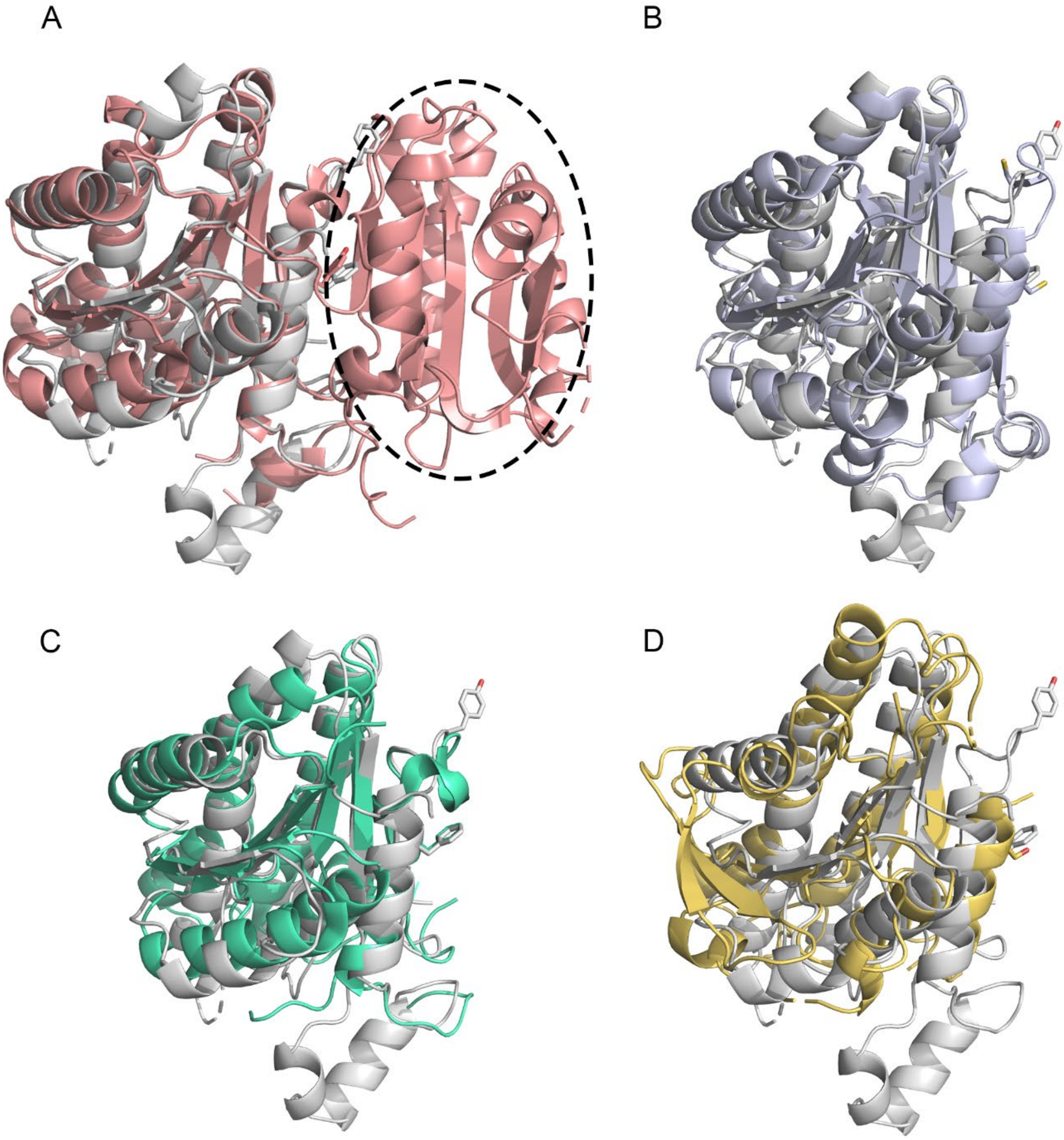

Figure S5. The superposition of FabG structures that are not conserved in the interface A. SpFabG is superposed with 3v1t (A), 3nyw (B), 5b1y (C), and 4fda (D) structures, respectively, showing that these four proteins are somewhat exceptional in conserved interface A. Flavodoxin type domain was circled by dotted lines.

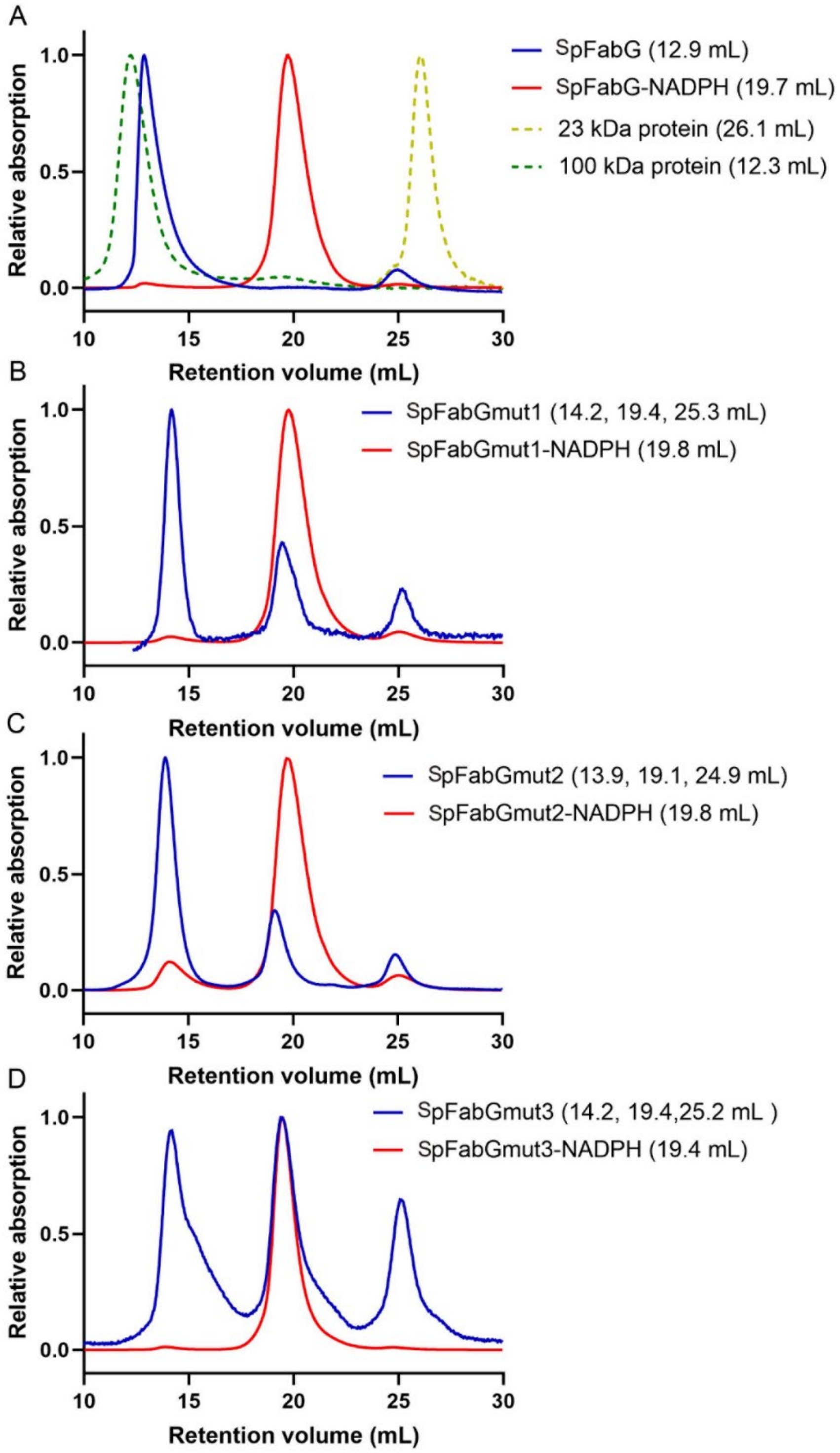

Figure S6. The existence forms of the wild-type and mutant SpFabG in solution were identified by analytical size-exclusion chromatography. (A)The wild-type SpFabG primarily exists as tetramers and, in the presence of NADPH, mainly exhibits dimer. Mutants with active triads (B) and interface A mutations (C-D) predominantly remain as tetramers in solution but show an increased proportion of dimers and monomers. In the presence of NADPH, these mutants primarily exist in the dimeric form. Proteins with molecular weights of 23 kDa and 100 kDa were used as molecular weight reference. The peaks located approximately at 14, 19, and 25 mL correspond to the tetrameric, dimeric, and monomeric states of SpFabG, respectively.

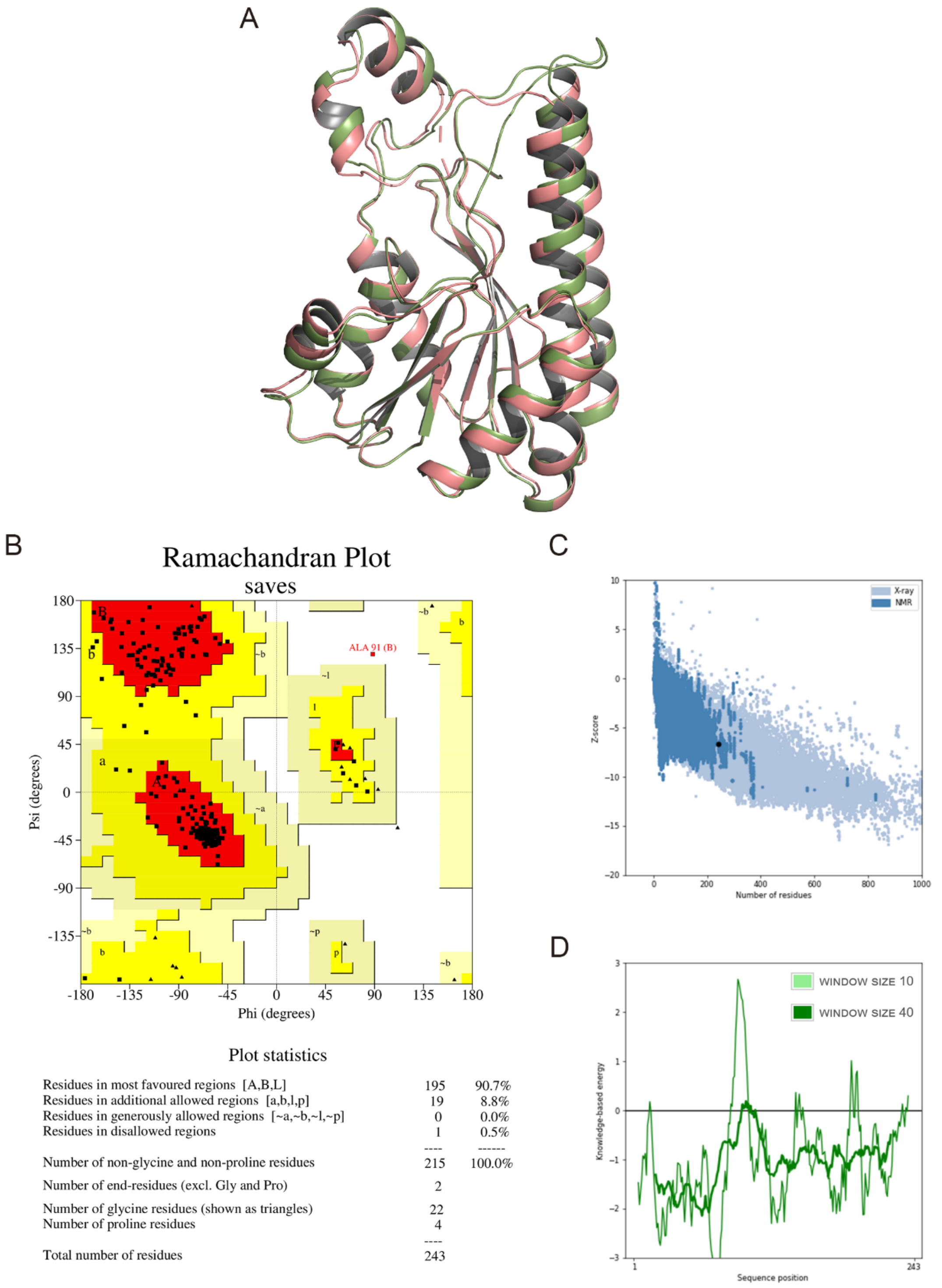

Figure S7. Structural information visuals of SpFabG chimeric model and model validation analysis. (A) Superimposed 3D structures of the chimeric model (green) and SpFabG X-ray structure template (red) (RMSD = 0.411 Å). (B) The Ramachandran plot and residue evaluations based on torsion angles. (C-D) ProSA plots. which compare a calculated Z score for the SpFabG homology model to that of protein structures experimentally determined by NMR spectroscopy and show the energy density levels across residues measured by two different window sizes.

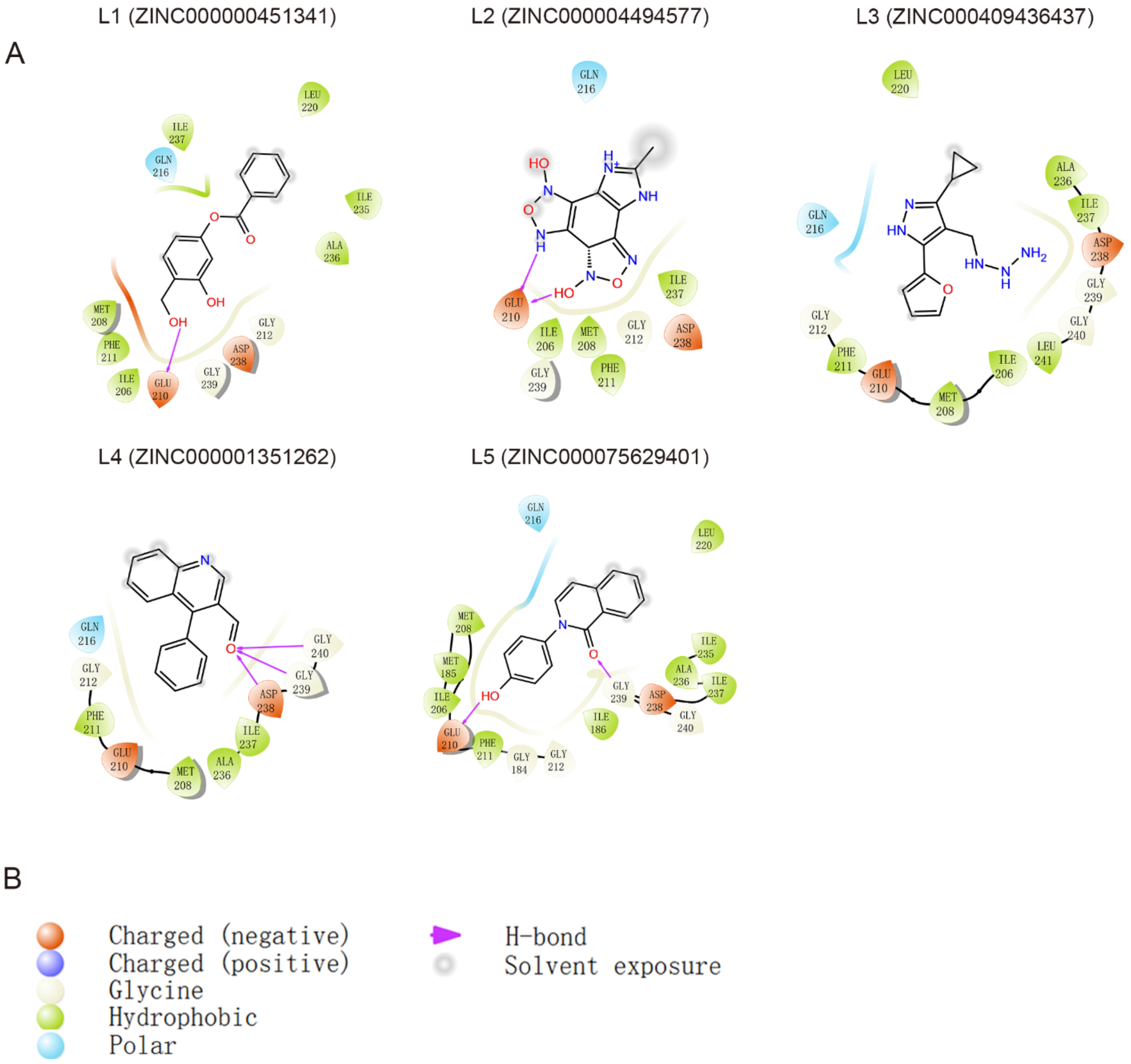

Figure S8. The 2D Schrodinger Maestro shows the top 5 ligands with ideal binding energies when docked with SpFabG using Glide (A). The Maestro legend (B).

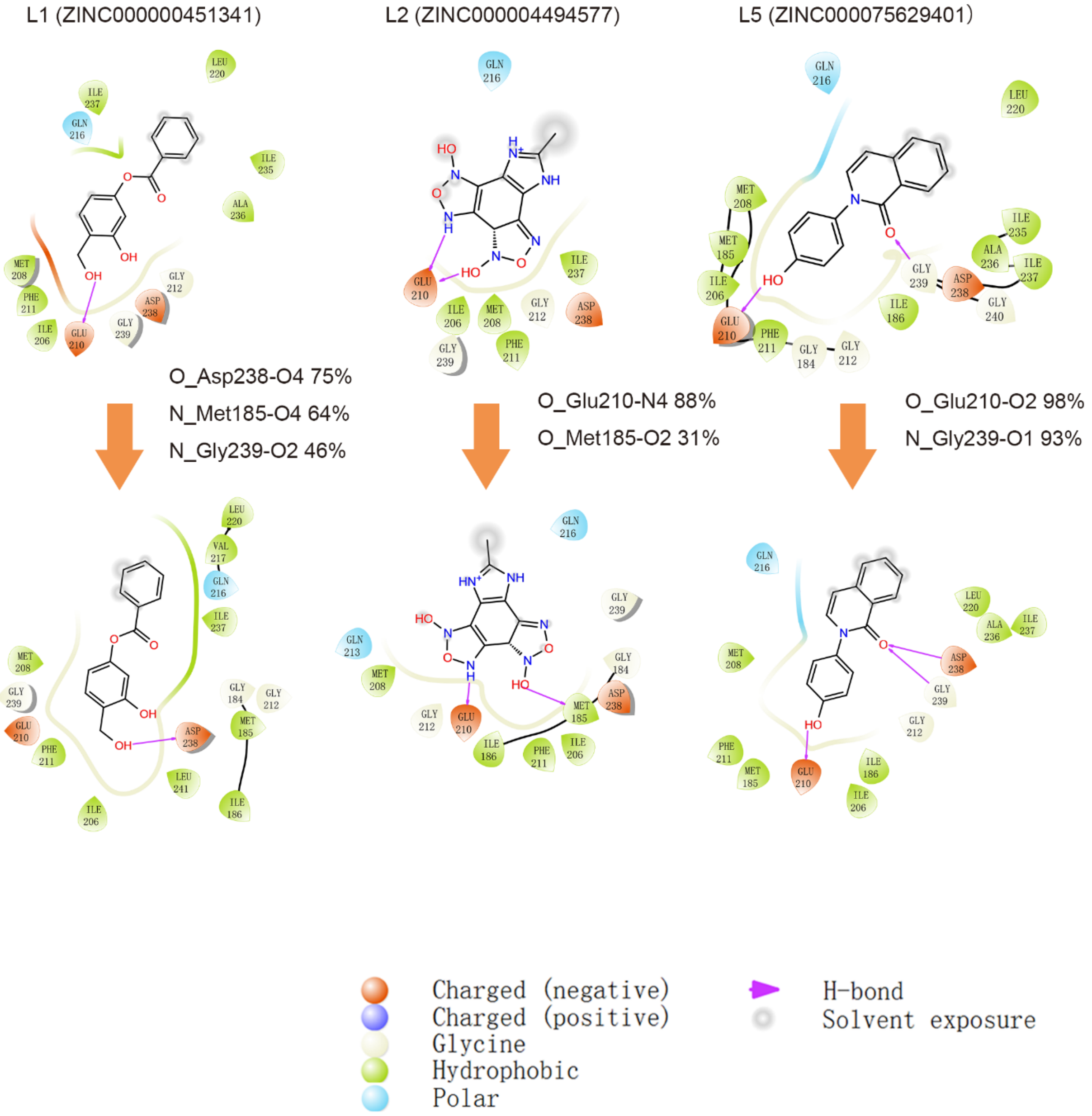

Figure S9. 2D Schrödinger Maestro visuals of the final state of the non-covalent bonds made with SpFabG by Lx from the initial post-docking pose after the 200 ns MD simulation.

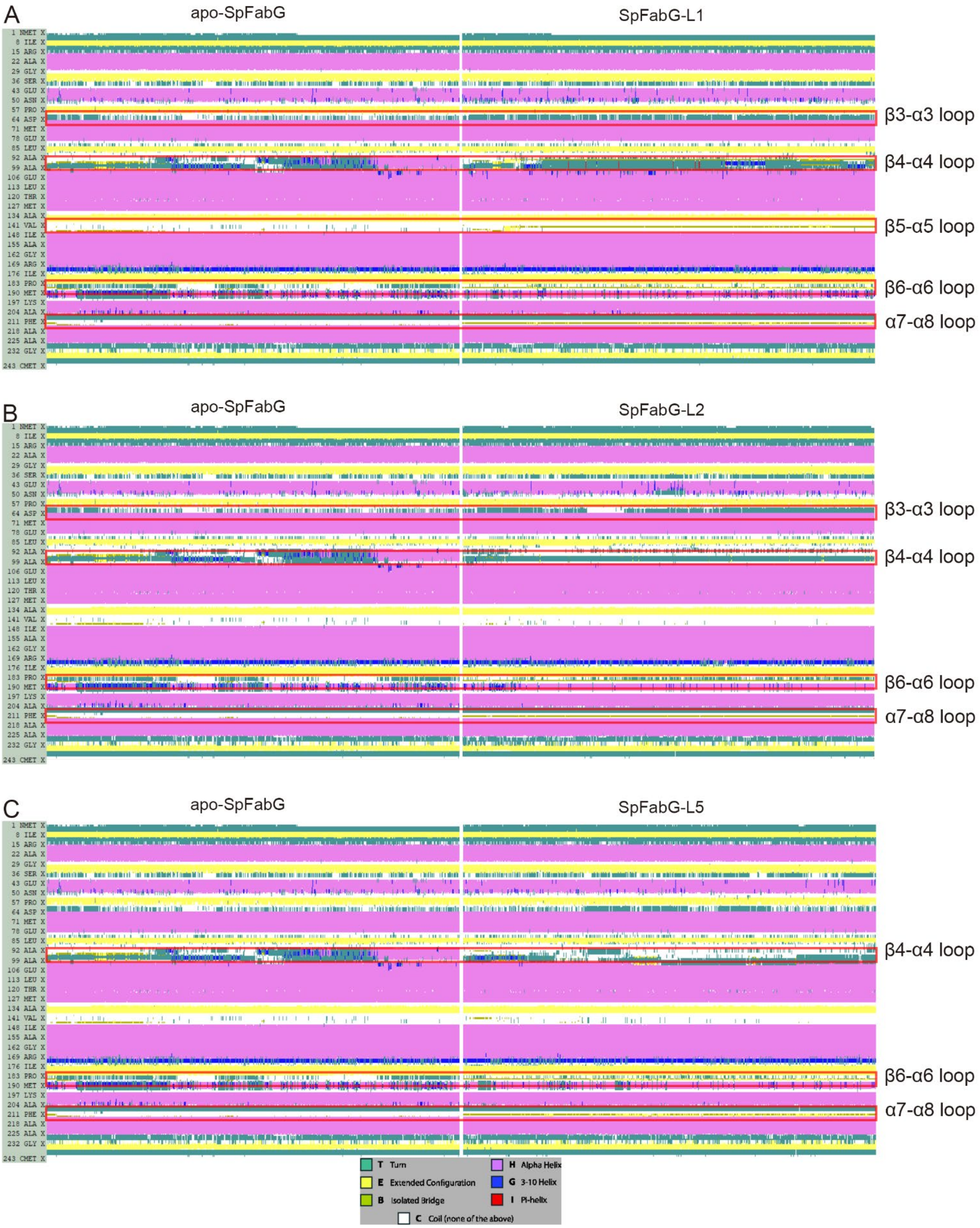

Figure S10. Timeline plot of apo-SpFabG and SpFabG-Lx. The areas indicated by red box are loop region surrounding the NADPH binding pocket. It has been observed that upon binding of the inhibitors, allosteric regulation occurs, modulating the secondary structure of these loop regions.
